# A versatile engineered extracellular vesicle platform simultaneously targeting and eliminating senescent stromal cells and tumor cells to promote tumor regression

**DOI:** 10.1186/s12951-024-02361-3

**Published:** 2024-03-11

**Authors:** Liangzhi Gong, Zhengsheng Chen, Kai Feng, Lei Luo, Juntao Zhang, Ji Yuan, Yajing Ren, Yang Wang, Xianyou Zheng, Qing Li

**Affiliations:** Institute of Microsurgery on Extremities, Department of Orthopaedics, Shanghai Sixth People’s Hospital Affiliated to Shanghai Jiao Tong University School of Medicine, Shanghai, 200233 China

**Keywords:** Small extracellular vesicles, Cellular senescence, siRNA, Stromal cell, Chemotherapy

## Abstract

Chemotherapy is an important therapeutic approach for malignant tumors for it triggers apoptosis of cancer cells. However, chemotherapy also induces senescence of stromal cells in the tumor microenvironment to promote tumor progression. Strategies aimed at killing tumor cells while simultaneously eliminating senescent stromal cells represent an effective approach to cancer treatment. Here, we developed an engineered Src-siRNA delivery system based on small extracellular vesicles (sEVs) to simultaneously eliminate senescent stromal cells and tumor cells for cancer therapy. The DSPE-PEG-modified urokinase plasminogen activator (uPA) peptide was anchored to the membranes of induced mesenchymal stem cell-derived sEVs (uPA-sEVs), and Src siRNA was loaded into the uPA-sEVs by electroporation (uPA-sEVs-siSrc). The engineered uPA-sEVs-siSrc retained the basic sEVs properties and protected against siSrc degradation. uPA peptide modification enhanced the sEVs with the ability to simultaneously target doxorubicin-induced senescent stromal cells and tumor cells. Src silencing by uPA-sEVs-siSrc induced apoptosis of both senescent stromal cells and tumor cells. The uPA-sEVs-siSrc displayed preferential tumor accumulation and effectively inhibited tumor growth in a tumor xenograft model. Furthermore, uPA-sEVs-siSrc in combination with doxorubicin significantly reduced the senescence burden and enhanced the therapeutic efficacy of chemotherapy. Taken together, uPA-sEVs-siSrc may serve as a promising therapy to kill two birds with one stone, not only killing tumor cells to achieve remarkable antitumor effect, but also eliminating senescent cells to enhance the efficacy of chemotherapeutic agent in tumor regression.

**Graphical Abstract:**

**Figure:**
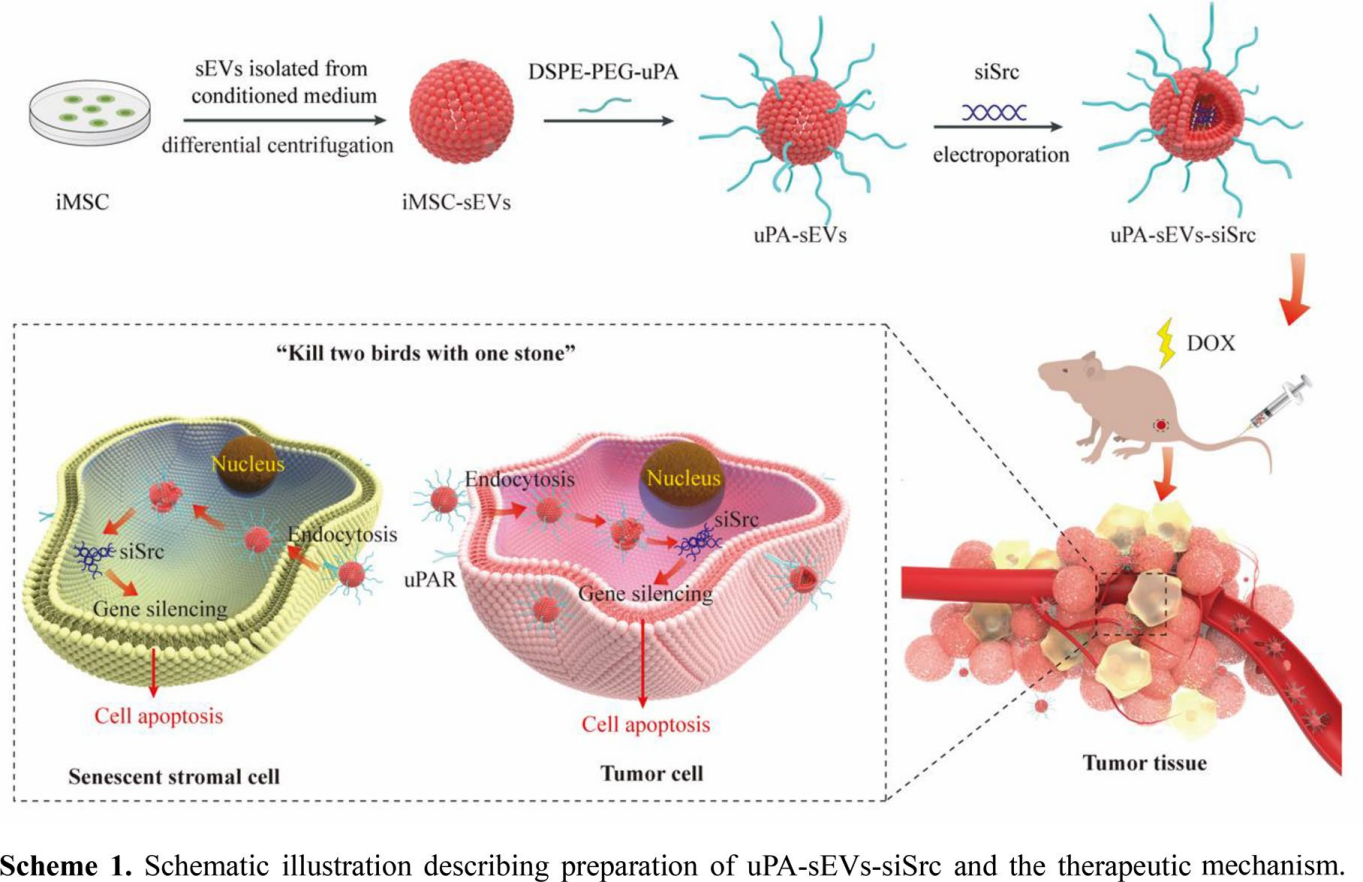

**Supplementary Information:**

The online version contains supplementary material available at 10.1186/s12951-024-02361-3.

## Introduction

Cancer is a major cause of death and public health problem worldwide [1]. Solid tumors have been considered as a complex “organ " composed of tumor cells and stromal cells [2, 3]. The crosstalk between stromal cells and tumor cells plays an important role in promoting tumor progression [3]. Chemotherapy is the most widely used cancer treatment option because of the high cytotoxicity of drugs such as doxorubicin (DOX) and cisplatin against cancer cell [4]. However, despite the efficacy of chemotherapy in inducing malignant cell apoptosis, stromal cells (e.g., fibroblasts, endothelial cells) in the tumor microenvironment are also susceptible to therapy-induced damage as part of the side effects of anticancer agents [5, 6]. Cellular senescence is a cellular state of irreversible proliferative arrest induced by various types of stress, including chemotherapy-induced stress [5]. Emerging evidence supports the role of chemotherapy-induced senescent stromal cells as an accomplice in the growth of a variety of solid tumors. For example, doxorubicin (DOX)-induced senescent human umbilical vein endothelial cells increase the aggressiveness of breast cancer cells by secreting CXCL11 [7]. Anticancer chemotherapeutics promote senescent phenotype in stromal fibroblasts, sustaining the invasive and clonogenic potential of both prostate and ovarian cancer cells [8]. Palbociclib-induced senescent fibroblasts significantly promote melanoma growth [9]. Due to the critical role of senescent stromal cells in tumor progression, several studies have reported that the use of either genetic approaches to clear p16^INK4A^-expressing cells or small molecule inhibitors (e.g., ABT-263) that selectively induce apoptosis of senescent stromal cells can significantly improve the efficacy of chemotherapy and lead to an increased healthy lifespan in tumor-bearing mice [10, 11]. Thus, therapeutic strategies aimed at killing tumor cells and simultaneously eliminating senescent stromal cells hold great potential for improving the therapeutic effect against cancer.

Src is a proto-oncogene tyrosine protein kinase that belongs to a family of non-receptor tyrosine kinases [12]. Hyper-activated of Src is found in a wide range of cancers, including lung [13], breast [14], pancreatic [15], colorectal [16], and prostate cancers [17] and is involved in the regulation of multiple tumor processes, such as cell proliferation, tumorigenesis, migration and resistance to radiotherapy and chemotherapy [18]. Moreover, Src is also involved in the regulation of cellular senescence. Gorospe et al. reported that Src-mediated activation of p38 critically promotes the senescence of fibroblasts with low DNA damage [19]. Lee et al. demonstrated that integrin α6β4-Src-AKT signaling induces cellular senescence by counteracting apoptosis in irradiated tumor cells and tissues [20]. Therefore, targeting Src may not only inhibit tumor cell proliferation but also counteract stromal cell senescence.

Small interfering RNA (siRNA)-based gene therapy enables the selective silencing of specific genes, providing a precise and effective treatment strategy for various diseases [21, 22]. The FDA approved the first siRNA therapeutic for the treatment of transthyretin-mediated amyloidosis [23]. Currently, several siRNA-based gene therapies are in clinical trials for a wide variety of diseases including cancers [24, 25]. As free siRNA can hardly cross the plasma membrane and is subject to degradation by ubiquitous ribonucleases, effective siRNA delivery systems, including polymer- or lipid-based carriers, are used for the application of siRNA therapeutics [26]. Recently, small extracellular vesicles (sEVs) have attracted considerable interest for therapeutic siRNA delivery [27–29]. sEVs are 30- to 200- nm diameter natural lipid vesicles with low-toxicity, low immunogenicity and can easily cross bio-membranes to enter cells, making sEVs a stable and suitable candidate for siRNA delivery [30, 31]. Mesenchymal stem cells (MSCs) derived sEVs have been widely used for delivery of siRNA [32, 33]. Remarkably, a new subtype of MSCs, iMSCs (derivates of induced pluripotent stem cells (iPSCs)) have attracted much attention in recent years. Since iPSCs possess unlimited proliferation ability, billions of iMSCs can be generated from iPSCs continuously, avoiding the invasive collection procedures required for conventional MSCs and ensuring the source stability of sEVs [34]. In addition, autologous iPSC derived MSCs or their sEVs can be used for treatment without causing ethical problems or immunological rejection, facilitating future clinical application [35]. Therefore, iMSC-sEVs were selected to deliver siRNA in the present study.

Targeted delivery of siRNA could increase the dose in the relevant tissue while reducing side-effect to other tissues [24]. Various approaches have been used for sEVs surface modification to increase targeted delivery, including genetic engineering, covalent modification, and non-covalent modification [36–39]. Urokinase plasminogen activator receptor (uPAR) has been identified as a cell surface protein that is highly expressed in several malignant tumors including lung, breast, pancreatic and colon cancer [40]. Accordingly, several studies have reported that uPAR-targeted nanoplatforms modified by the uPA peptide possessed great potential in enhancing tumor targeting, improving delivery efficiency, reducing drug toxicity, and in multimodal synergistic antitumor applications [41–44]. In addition, uPAR has also been reported as a cell surface protein that is broadly upregulated during senescence [45]. Amor et al. demonstrated that uPAR-targeted CAR T cells can specifically and efficiently eliminate senescent cells to treat lung cancer and liver fibrosis [45]. Therefore, we propose that uPAR may be a suitable candidate target for both senescent stromal cells and tumor cells.

Herein, we constructed an engineered Src-siRNA delivery system based on iMSC-sEVs. The DSPE-PEG-modified uPA peptide was anchored on sEVs membrane (uPA-sEVs), and then Src siRNA (siSrc) was loaded into uPA-sEVs via electroporation to construct an engineered platform (uPA-sEVs-siSrc). The results showed that uPA-sEVs-siSrc could simultaneously target and induce apoptosis of senescent stromal cells and tumor cells. In vivo studies showed that uPA-sEVs-siSrc alone can reduce tumor size, and combined treatment of uPA-sEVs-siSrc with the senescence-inducing chemotherapy DOX can significantly reduce senescence burden and halt tumor progression in a tumor xenograft model of human breast cancer. Taken together, these data demonstrate for the first time that uPA-sEVs-siSrc may serve as a promising therapy to simultaneously target and eliminate senescent stromal cells and tumor cells, and to enhance the efficacy of chemotherapeutic agents in tumor regression.

## Results and discussion

### Preparation and characterization of uPA-sEVs-siSrc

First, the iMSCs used in this study were characterized by flow cytometry analysis. The result showed that iMSCs were positive for the surface antigens CD44, CD29, CD146 and CD105, and negative for CD45, CD133 and HLA-DR (Figure S1A). sEVs were then isolated from iMSC-derived conditioned media and characterized in terms of morphology and particle size. The purified iMSC-sEVs displayed a cup-shaped morphology with a size around 100 nm, as shown by TEM (Figure S1B). NanoFCM analysis revealed that the majority of iMSC-sEVs distributed between 40 and 125 nm, and the mean diameter was 68.25 ± 15.70 nm (Figure S1C). These results indicate that sEVs were successfully isolated from the conditioned medium of iMSCs.

**Fig. 1:**
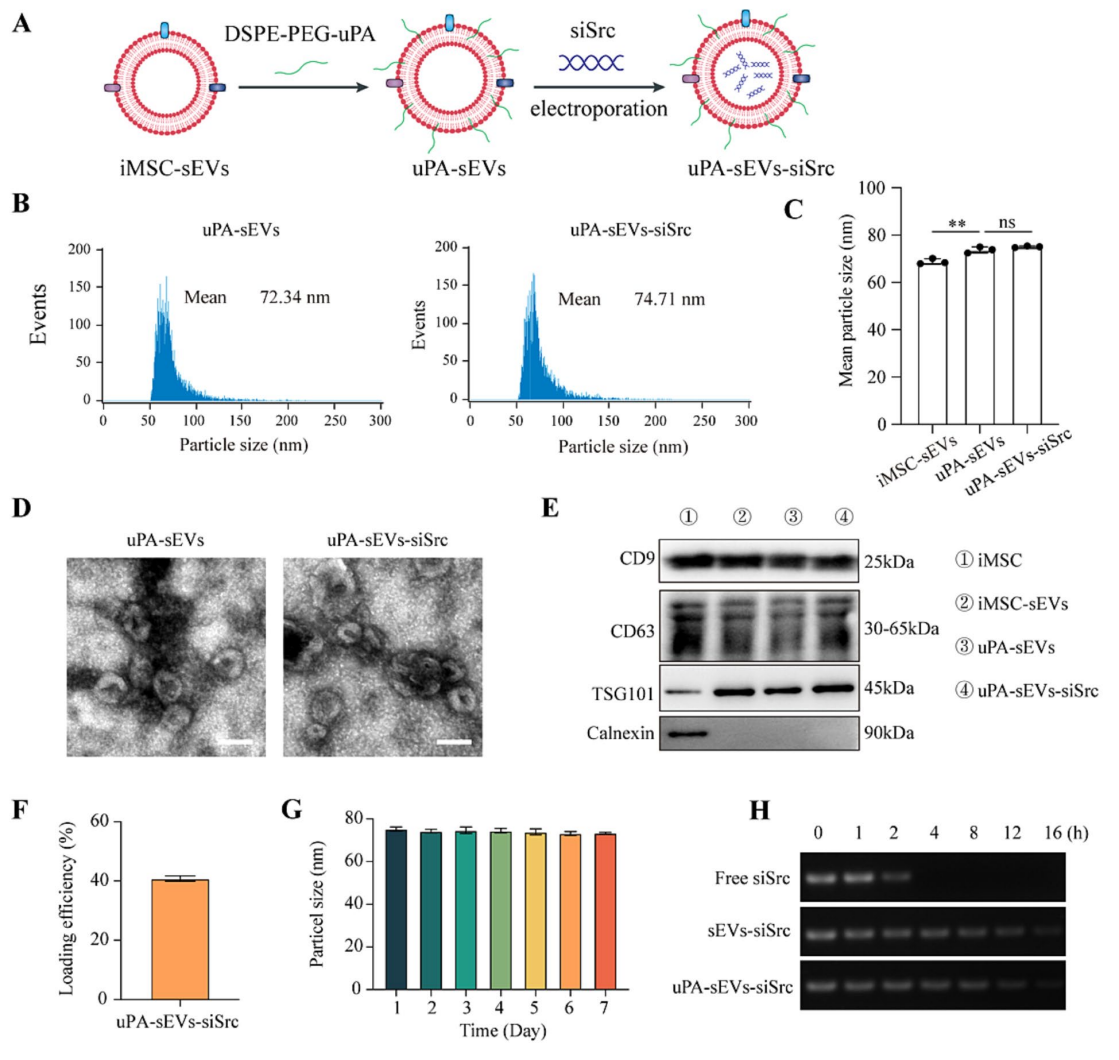
Preparation and characterization of uPA-sEVs-siSrc. (**A**) Schematic illustration describing the design of uPA-sEVs-siSrc. (**B**) Size distribution of uPA-sEVs and uPA-sEVs-siSrc measured by nanoFCM. (**C**) Mean particle size of iMSC-sEVs, uPA-sEVs and uPA-sEVs-siSrc measured by nanoFCM. (**D**) Representative TEM images of uPA-sEVs and uPA-sEVs-siSrc. Scale bar = 200 nm. (**E**) Western blot analysis of iMSC, iMSC-sEVs, uPA-sEVs and uPA-sEVs-siSrc. (**F**) siRNA loading efficiency of uPA-sEVs-siSrc determined by a microplate reader. (**G**) Stability of dynamic diameter of uPA-sEVs-siSrc. (**H**) Serum stability of free siSrc, sEVs-siSrc and uPA-sEVs-siSrc analyzed by gel electrophoresis

Next, iMSC-sEVs were incubated with the DSPE-PEG modified uPA peptide (uPA-sEVs), and siSrc was then encapsulated into uPA-sEVs by electroporation to construct uPA-sEVs-siSrc (Fig. 1A). NanoFCM analysis showed that the mean diameter of uPA-sEVs and uPA-sEVs-siSrc was 72.34 ± 15.97 nm and 74.71 ± 16.44 nm, respectively (Fig. 1B). As shown in Fig. 1C, DSPE-PEG-uPA modification on sEVs can slightly increase the particle size, but there is no significant difference in particle size between uPA-sEVs and uPA-sEVs-siSrc (Fig. 1C). To further confirm the success of uPA modification on sEVs and to evaluate the modification efficiency, the FITC-labelled uPA-sEVs and uPA-sEVs-siSrc were analyzed by nanoFCM. As shown in Figure S2A, B, the percentage of FITC-positive particles in FITC-DSPE-PEG-uPA-sEVs and FITC-DSPE-PEG-uPA-sEVs-siSrc was 73.97 ± 0.99% and 70.7 ± 3.81%, respectively. These results indicated that iMSC-sEVs were successfully modified with the uPA peptide and that the loading of siSrc did not affect peptide labelling. The morphology of uPA-sEVs and uPA-sEVs-siSrc were characterized by TEM, and both exhibited a typical cup-like morphology (Fig. 1D). Western blot analysis showed that uPA-sEVs and uPA-sEVs-siSrc still retained the protein markers of sEVs including CD9, CD63 and TSG101, but negative for the endoplasmic reticulum marker-Calnexin (Fig. 1E). The average zeta potential of the iMSC-sEVs, uPA-sEVs and uPA-sEVs-siSrc was − 23.49 ± 1.32 mV, -19.80 ± 0.44 mV and − 18.40 ± 1.73 mV, respectively (Figure S2C). Compared to the iMSC-sEVs group, the modification of the uPA peptide slightly changed the zeta potential of sEVs. These results indicate that the modification of uPA peptide and the loading of siSrc via electroporation do not alter the physical properties of the sEVs. We next examined the siRNA loading efficiency as previously described [46]. As shown in Fig. 1F, siRNA was incorporated into uPA-sEVs with a high loading efficiency up to 45%. To determine the stability of uPA-sEVs-siSrc, particle size of uPA-sEVs-siSrc was monitored by nanoFCM for 7 consecutive days. As shown in Fig. 1G, the hydrodynamic diameter of uPA-sEVs-siSrc was slightly changed, indicating that uPA-sEVs-siSrc had stable physical characteristics. Then, the sEVs protection of siRNA was studied by testing the siSrc levels after incubation with FBS for a series of time intervals. Free siSrc incubated with FBS was used as the control. As shown in Fig. 1H, electrophoresis bands of siSrc in uPA-sEVs and sEVs alone were still detectable at 12 h, while the bands of free siSrc solution disappeared at 2 h, indicating that encapsulation of siSrc in sEVs could protect siSrc from serum nuclease degradation.

### *In vitro* targeting capability of uPA-sEVs

To verify the targeting ability of sEVs modified by the uPA peptide, we first evaluated the expression of uPAR in proliferative and senescent stromal cells (HFF-1 and HMEC-1), and tumor cells (A549 and MDA-MB-231). To establish DOX-induced senescent HFF-1 and HMEC-1 cells, HFF-1 and HMEC-1 cells were treated with 100 nM DOX for 3 days, then cultured under non-DOX medium for 4 days and measured several well-established senescence markers (SA-β-gal and P21) as previously described [19]. As shown in Figure S3A, DOX treatment increased SA-β-gal activity in HFF-1 and HMEC-1 cells. The expression levels of senescence markers P21 were also markedly elevated by DOX treatment (Figure S3B). These results showed that HFF-1 and HMEC-1 cells were successfully induced senescence by DOX treatment. Then, the expression of uPAR in the proliferative and senescent stromal cells (HFF-1 and HMEC-1), and tumor cells (A549 and MDA-MB-231) was measured by IF staining. As shown in Figure S3C, higher levels of uPAR expression were observed on the membrane of senescent HFF-1 and HMEC-1 cells compared to proliferative HFF-1 and HMEC-1 cells (Figure S3C). Moreover, high levels of uPAR expression were also observed on the membrane of human lung cancer A549 and breast cancer MDA-MB-231 cells (Figure S3C).

**Fig. 2:**
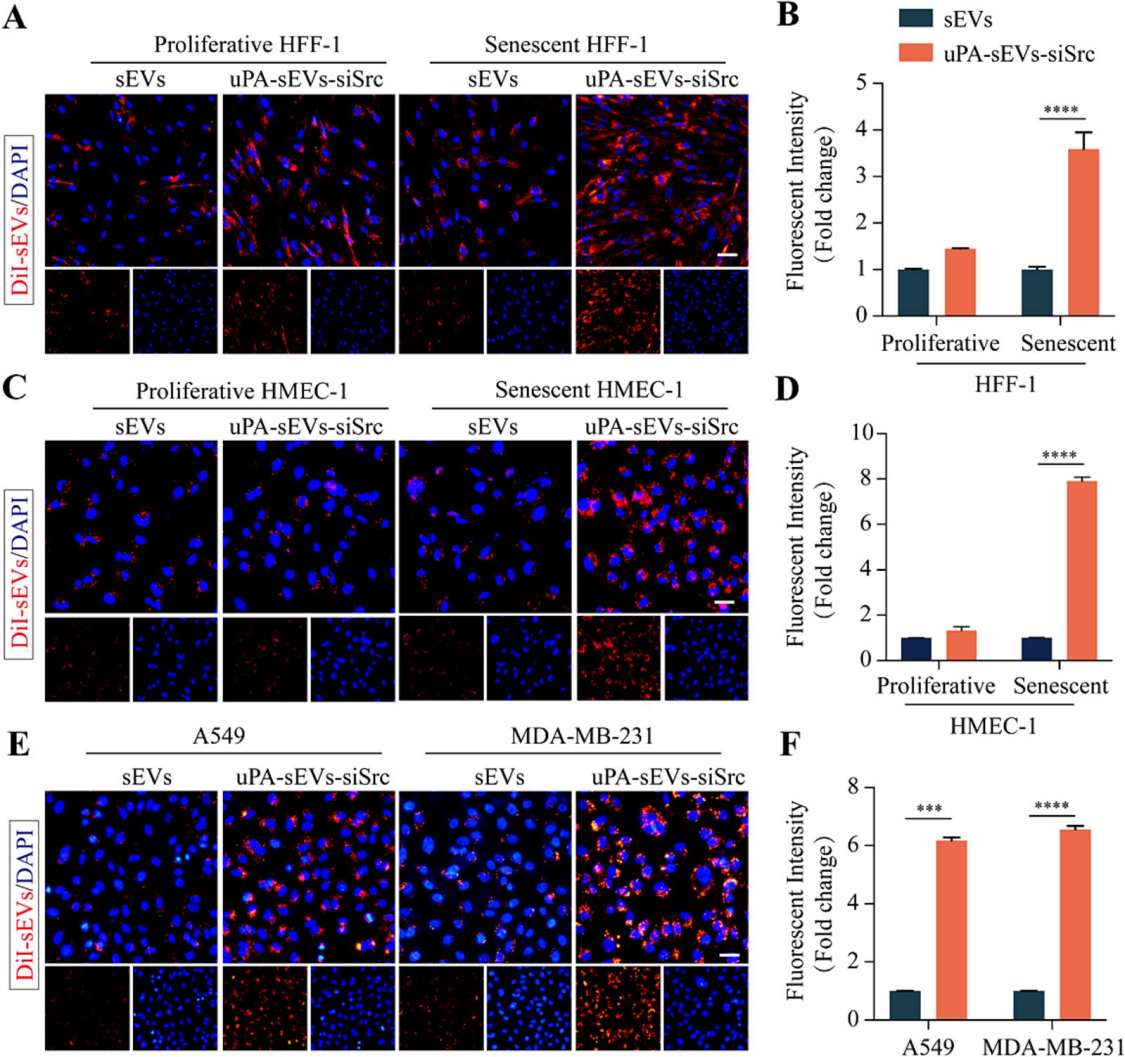
Targeting capability of uPA-sEVs-siSrc in vitro. (**A**) Representative images of proliferative and senescent HFF-1 cells incubated with DiI-labelled sEVs and uPA-sEVs-siSrc for 4 h. Scale bar = 50 μm. (**B**) Mean fluorescence intensities from (**A**). (**C**) Representative images of proliferative and senescent HMEC-1 cells incubated with DiI-labelled sEVs and uPA-sEVs-siSrc for 4 h. Scale bar = 50 μm. (**D**) Mean fluorescence intensities from (**C**). (**E**) Representative images of A549 and MDA-MB-231 cells incubated with DiI-labelled sEVs and uPA-sEVs-siSrc for 4 h. Scale bar = 50 μm. (**F**) Mean fluorescence intensities from (**E**). Data are displayed as the mean ± SD (*n* = 3). ^***^p<0.001; ^****^p<0.0001

Next, we examined the cellular uptake of DiI fluorescent dye-labeled sEVs (DiI-sEVs) or uPA-sEVs (DiI-uPA-sEVs) in the proliferative and senescent HFF-1 and HMEC-1 cells and in the tumor A549 and MDA-MB-231 cells in *vitro*. As shown in Fig. 2A-D, the fluorescence intensity was much higher in senescent HFF-1 cells (Fig. 2A, B) and HMEC-1 cells (Fig. 2C, D) in DiI-uPA-sEVs group compared to DiI-sEVs group. We also evaluated the cellular uptake of DiI-sEVs or DiI-uPA-sEVs in the A549 and MDA-MB-231 cells. As shown in Fig. 2E, F, DiI-uPA-sEVs group showed much higher fluorescence intensity in A549 cells (Fig. 2E) and MDA-MB-231 cells (Fig. 2F) compared to DiI-sEVs group. Moreover, quantitative analysis of cellular uptake of DiI-sEVs and DiI-uPA-sEVs was also assessed by flow cytometry. As shown in Figure S3, the DiI-uPA-sEVs group had a much higher uptake efficiency in senescent stromal cells (HFF-1 and HMEC-1 cells) and tumour cells (A549 and MDA-MB-231 cells) compared to the DiI-sEVs group. However, there was no significant difference in uptake efficiency in proliferative stromal cells (Figure S4). These results indicated that uPA peptide modification on iMSC-sEVs could effectively enhance the cellular uptake of sEVs in senescent stromal cells and tumor cells.

The lysosomal escape of uPA-sEVs-siSrc in stromal cells and tumor cells was tracked via fluorescent colocalization. uPA-sEVs-siSrc and lysosome were labelled with DiI (red) and lysosome marker LysoTracker (green) respectively. Proliferative and senescent stromal cells (HFF-1 and HMEC-1 cells), as well as tumor cells (A549 and MDA-MB-231 cells) were incubated with uPA-sEVs-siSrc for 2 h, 8 and 16 h, respectively. After 2 h incubation, the signals of DiI and LysoTracker overlay, reflecting the entrance of uPA-sEVs-siSrc in lysosome; after 8 h, signals of DiI and LysoTracker partially separated, indicating that uPA-sEVs-siSrc began to escape from lysosome; after 16 h, signals of DiI and LysoTracker completely separated, showing the successful lysosomal escape of uPA-sEVs-siSrc (Figure S5). These results demonstrated the process of uPA-sEVs-siSrc uptake and intracellular lysosomal escape.

### uPA-sEVs-siSrc treatment effectively induces apoptosis of senescent stromal cells

**Fig. 3:**
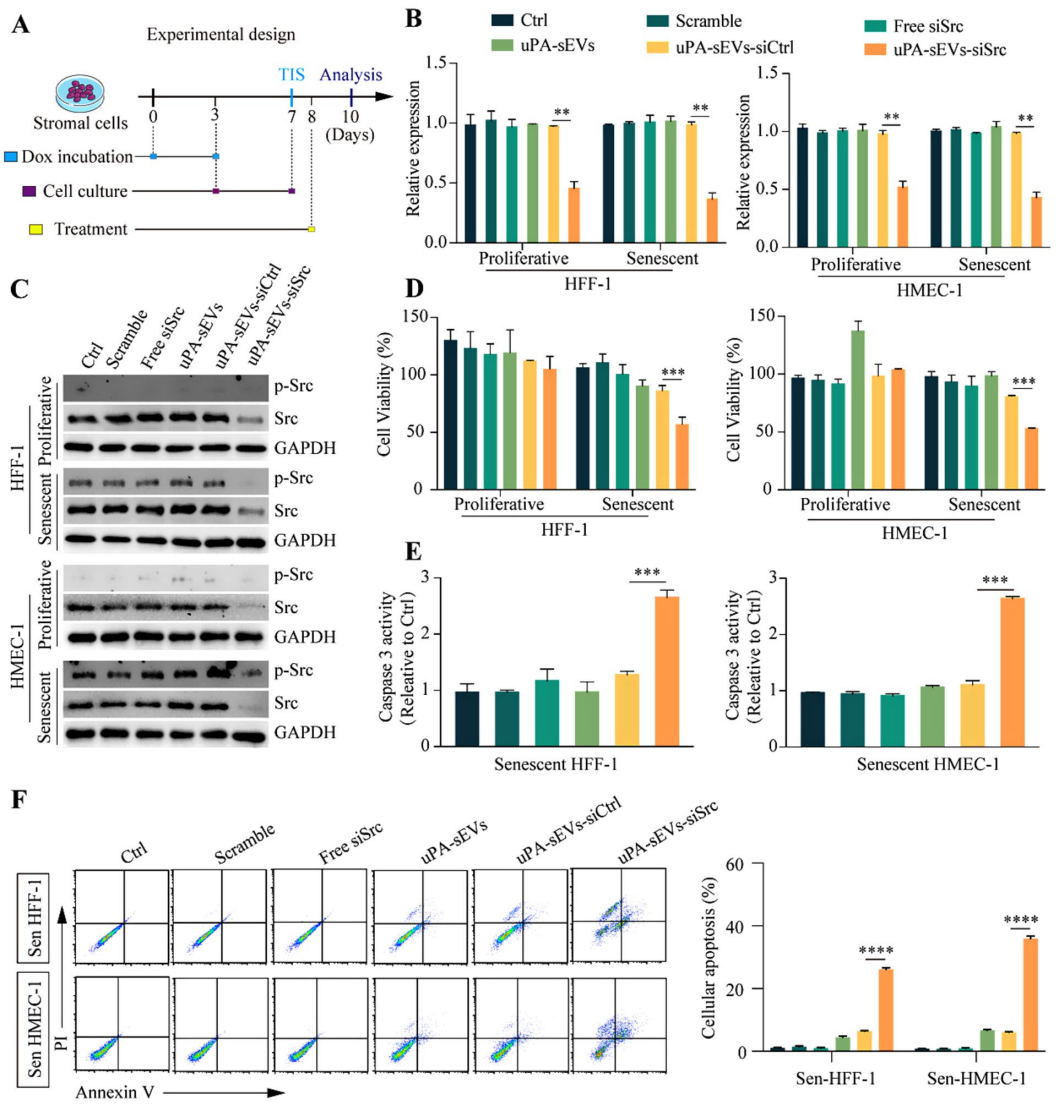
uPA-sEVs-siSrc induce apoptosis of senescent stromal cells in vitro. (**A**) Schematic illustration describing the experimental design in vitro. (**B**) mRNA expression of Src in proliferative and senescent HFF-1 and HMEC-1 cells with different treatments determined by RT-qPCR respectively. (**C**) Protein levels of p-Src and Src in proliferative and senescent HFF-1 and HMEC-1 cells with different treatments determined by western blotting. (**D**) Cell viability analysis of proliferative and senescent HFF-1 and HMEC-1 cells with different treatment for 48 h. (**E**) Caspase 3 activity of senescent HFF-1 and HMEC-1 cells with different treatments. (**F**) Flow cytometric examination and quantitative analysis of senescent HFF-1 and HMEC-1 cells apoptosis with different treatments. Data are displayed as the mean ± SD (*n* = 3). ^**^p<0.01; ^***^p<0.001; ^****^p<0.0001

To investigate whether uPA-sEVs-siSrc could efficiently deliver siSrc to proliferative and senescent stromal cells, the proliferative and DOX-induced senescent stromal cells were treated with PBS, scrambled siRNA, free siSrc, uPA-sEVs, uPA-sEVs-siCtrl and uPA-sEVs-siSrc, respectively. After 48 h treatment, the expression of the target gene Src was quantified at the mRNA and protein levels by RT-qPCR and western blot assays, respectively (Fig. 3A). As shown in Fig. 3B, compared to PBS and scrambled siRNA groups, addition of free siSrc directly to the medium did not affect the mRNA expression of Src in proliferative and senescent HFF-1 and HMEC-1 cells. Whereas addition of uPA-sEVs-siSrc showed a remarkable inhibitory effect on the mRNA expression of Src with a knockdown efficiency of up to 50% in proliferative and senescent HFF-1 and HMEC-1 cells, compared to uPA-sEVs and uPA-sEVs-siCtrl. Then, the protein levels of total Src (Src) and phosphorylation-activated Src (p-Src-Y416) were examined in proliferative and senescent HFF-1 and HMEC-1 cells. As shown in Fig. 3C, p-Src was barely detectable in proliferating cells, but was highly expressed in senescent cells, indicating the activation of Src in senescent stromal cells. Compared to PBS and scrambled siRNA, free siSrc did not affect the protein expression of p-Src and Src in proliferative and senescent HFF-1 and HMEC-1 cells. uPA-sEVs-siSrc effectively silenced the expression of p-Src and total Src in proliferative and senescent HFF-1 and HMEC-1 cells, compared to uPA-sEVs and uPA-sEVs-siCtrl (Fig. 3C). These studies indicated that uPA-sEVs-siSrc can efficiently deliver siSrc into cells and silence Src expression in proliferative and senescent stromal cells.

**Fig. 4:**
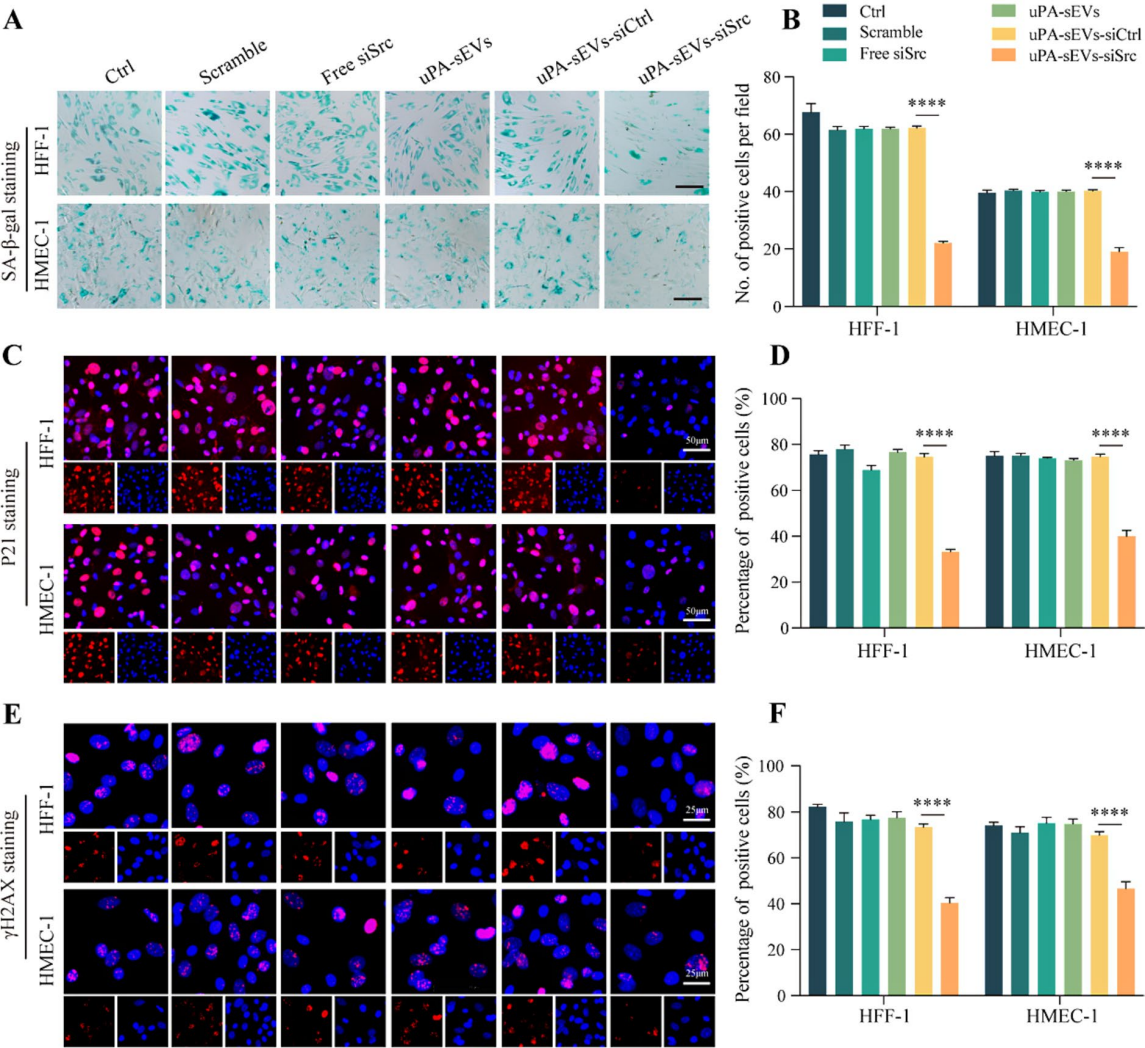
uPA-sEVs-siSrc effectively eliminate senescent stromal cells in vitro. (**A**, **B**) SA-β-gal staining (**A**) and quantitative analysis (**B**) of senescent HFF-1 and HMEC-1 cells with different treatments. Scale bar = 100 μm. (**C**, **D**) P21 staining (**C**) and quantitative analysis (**D**) of senescent HFF-1 and HMEC-1 cells with different treatments. Scale bar = 50 μm. (**E**, **F**) γH2AX staining (**E**) and quantitative analysis (**F**) of senescent HFF-1 and HMEC-1 cells with different treatments. Scale bar = 25 μm. Data are displayed as the mean ± SD (*n* = 3). ^****^p<0.0001

We next tested whether uPA-sEVs-siSrc could affect the proliferation and apoptosis of stromal cells. As shown in Fig. 3D, free siSrc did not affect the viability of senescent HFF-1 and HMEC-1 cells compared to PBS and scrambled siRNA, whereas uPA-sEVs-siSrc treatment dramatically reduced the viability of senescent HFF-1 and HMEC-1 cells compared to uPA-sEVs and uPA-sEVs-siCtrl. Analysis of caspase 3 activity also revealed that uPA-sEVs-siSrc treatment significantly increased caspase 3 activity in senescent stromal cells (Fig. 3E). Flow cytometric assay showed that apoptotic efficiency reached 26.1% in senescent HFF-1 cells and 35.9% in senescent HMEC-1 cells treated with uPA-sEVs-siSrc (Fig. 3F). Furthermore, several well-established senescence markers (SA-β-gal, P21 and γH2AX) were performed. As shown in Fig. 4A, B, free siSrc did not affect the number of SA-β-gal-positive HFF-1 and HMEC-1 cells compared to PBS, scrambled siRNA, whereas uPA-sEVs-siSrc treatment dramatically reduced the number of SA-β-gal-positive HFF-1 and HMEC-1 cells compared to uPA-sEVs and uPA-sEVs-siCtrl. Similarly, uPA-sEVs-siSrc treatment dramatically reduced the percentage of P21- and γH2AX-positive HFF-1 and HMEC-1 cells (Fig. 4C-F). These results suggest that uPA-sEVs-siSrc treatment can effectively induce apoptosis of senescent stromal cells.

### uPA-sEVs-siSrc treatment induces apoptosis of tumor cells

**Fig. 5:**
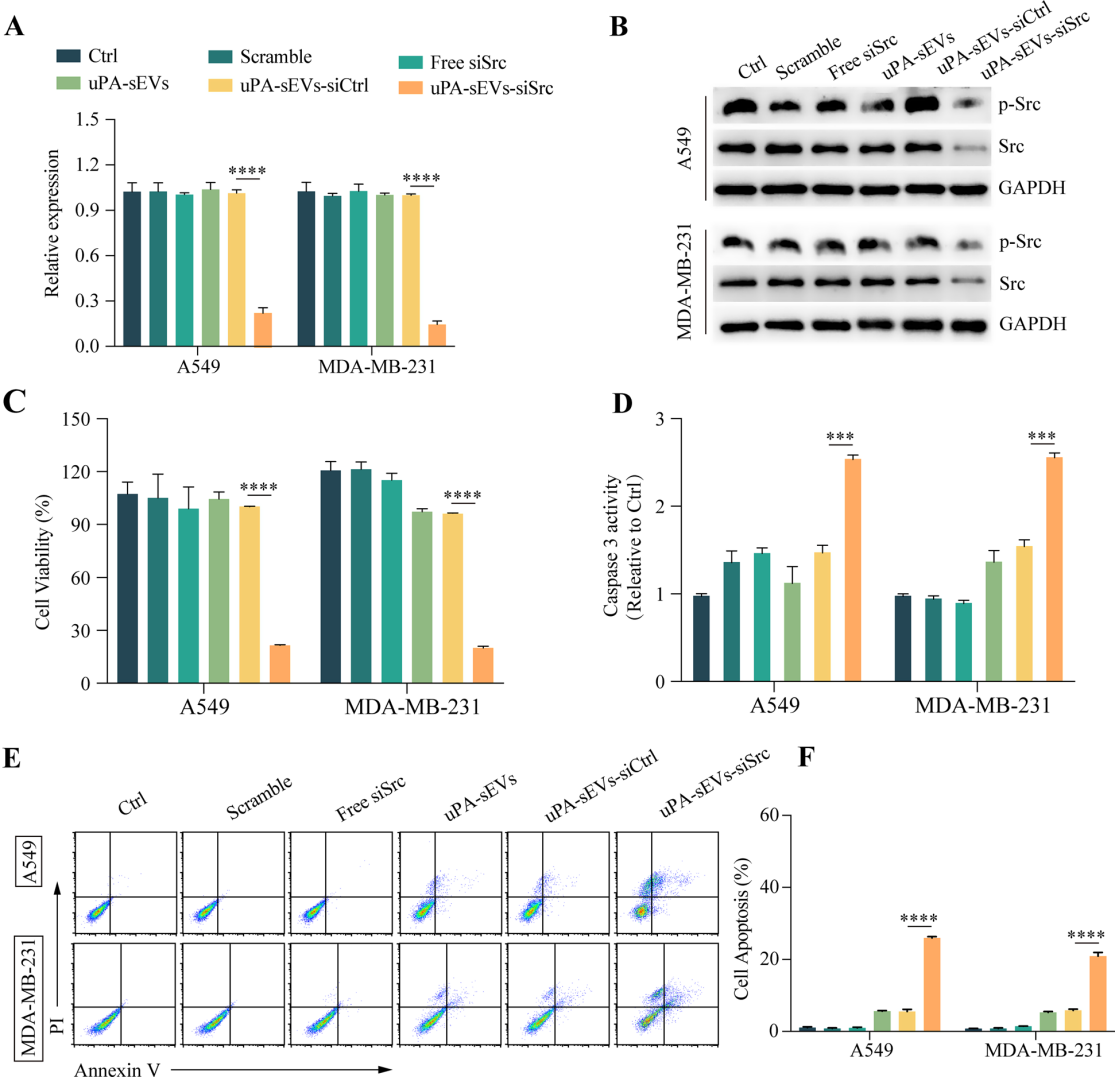
uPA-sEVs-siSrc induce apoptosis of tumor cells in vitro. (**A**) mRNA expression of Src in tumor cells with different treatments determined by RT-qPCR respectively. (**B**) Protein levels of p-Src and Src in tumor cells with different treatments determined by western blotting. (**C**) Cell viability analysis of tumor cells with different treatment for 48 h. (**D**) Caspase 3 activity of tumor cells after different treatments. (**E**, **F**) Flow cytometric examination (**E**) and quantitative analysis (**F**) of tumor cells apoptosis after different treatments. Data are displayed as the mean ± SD (*n* = 3). ^***^p<0.001; ^****^p<0.0001

Next, the effect of uPA-sEVs-siSrc treatment on tumor cell viability was assessed. First, we investigated gene silencing of Src in tumor cells (A549 and MDA-MB-231) in vitro. As shown in Fig. 5A, B, uPA-sEVs-siSrc treatment showed a remarkable inhibitory effect on the mRNA and protein expression of p-Src and Src in A549 and MDA-MB-231 cells, indicating effective gene silencing by uPA-sEVs-siSrc in tumor cells. Then, the ability of uPA-sEVs-siSrc to inhibit tumor cell growth was tested. As shown in Fig. 5C, free siSrc did not affect the viability of A549 and MDA-MB-231 cells compared to PBS and scrambled siRNA, whereas uPA-sEVs-siSrc treatment dramatically reduced the viability of A549 and MDA-MB-231 cells compared to uPA-sEVs and uPA-sEVs-siCtrl. Similarly, analysis of caspase 3 activity revealed that uPA-sEVs-siSrc significantly increased caspase 3 activity in A549 and MDA-MB-231 cells (Fig. 5D). A flow cytometric assay was also used to investigate the effect on tumor cells apoptosis. As shown in Fig. 5E, F, apoptotic efficiency reached 35.9% in A549 and 30.9% in MDA-MB-231 cells treated with uPA-sEVs-siSrc. These results demonstrate that uPA-sEVs-siSrc treatment can also effectively induce apoptosis of tumor cells. Moreover, flow cytometry assay showed that the apoptotic efficiency reached 32.01 ± 1.14% in A549 cells and 35.23 ± 1.0% in MDA-MB-231 cells treated with Dox alone, while the apoptotic efficiency reached 42.22 ± 1.42% in A549 cells and 52.96 ± 1.63% in MDA-MB-231 cells treated with Dox combined with uPA-sEVs-siSrc (Figure S6). These results indicate that uPA-sEVs-siSrc combined with Dox can further enhance the apoptosis of tumor cells.

### *In vivo* targeting capability of uPA-sEVs-siSrc

**Fig. 6:**
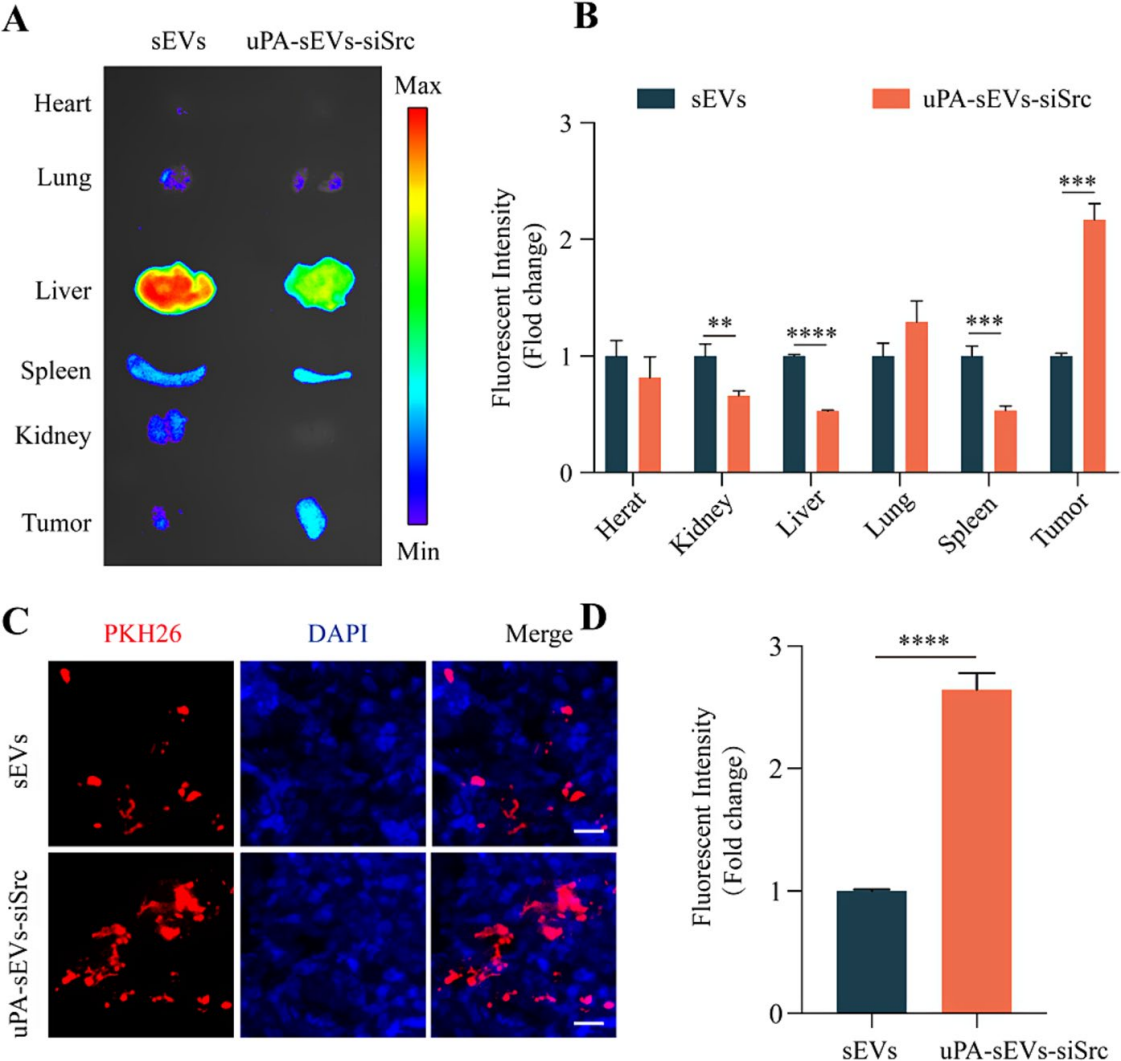
Targeting ability of uPA-sEVs-siSrc in vivo. (**A**) Ex vivo fluorescent images of the main organs (heart, lung, liver, spleen, kidney) and tumor at 8 h post-injection. (**B**) Quantitative biodistribution of PKH26-labelled sEVs and uPA-sEVs-siSrc in the main organs and tumors. (**C**) Biodistribution of PKH26-labelled sEVs and uPA-sEVs-siSrc in tumor sections. Nuclei were stained with DAPI. Scale bar = 20 μm. (**D**) Quantitative biodistribution of PKH26-labelled sEVs and uPA-sEVs-siSrc in the tumor sections. ^**^p<0.01; ^***^p<0.001; ^****^p<0.0001

Encouraged by the dual ability of uPA-sEVs-siSrc to target and induce apoptosis of senescent stromal cells and tumor cells in vitro, we next investigated the anti-tumor activity of uPA-sEVs- siSrc *in vivo.* To determine the cellular tropism of uPA-sEVs-siSrc in vivo, the biodistribution of PKH26-labelled sEVs or uPA-sEVs-siSrc was evaluated in MDA-MB-231 breast cancer tumor–bearing mice. As shown in Figure S7, a near-infrared signal was distributed in tumors over time. Ex vivo imaging of dissected organs (heart, liver, spleen, lung, and kidney) and tumors at 8 h after administration. As shown in Fig. 6A, B, PKH26-labelled uPA-sEVs-siSrc exhibited reduced distribution in major organs (heart, kidney, liver, and spleen) and enhanced tumor accumulation, with 3-fold higher fluorescence intensity than PKH26-labelled sEVs in tumors. Furthermore, the biodistribution of sEVs and uPA-sEVs-siSrc was further evaluated in tumor sections. As shown in Fig. 6C, D, uPA-sEVs-siSrc showed stronger red fluorescence in the tumor sections than sEVs. These results indicated that the uPA peptide modification on sEVs effectively enhanced the tumor targeting ability *in vivo*.

### uPA-sEVs-siSrc treatment promotes tumor regression and enhances chemotherapy efficiency *in vivo*

**Fig. 7:**
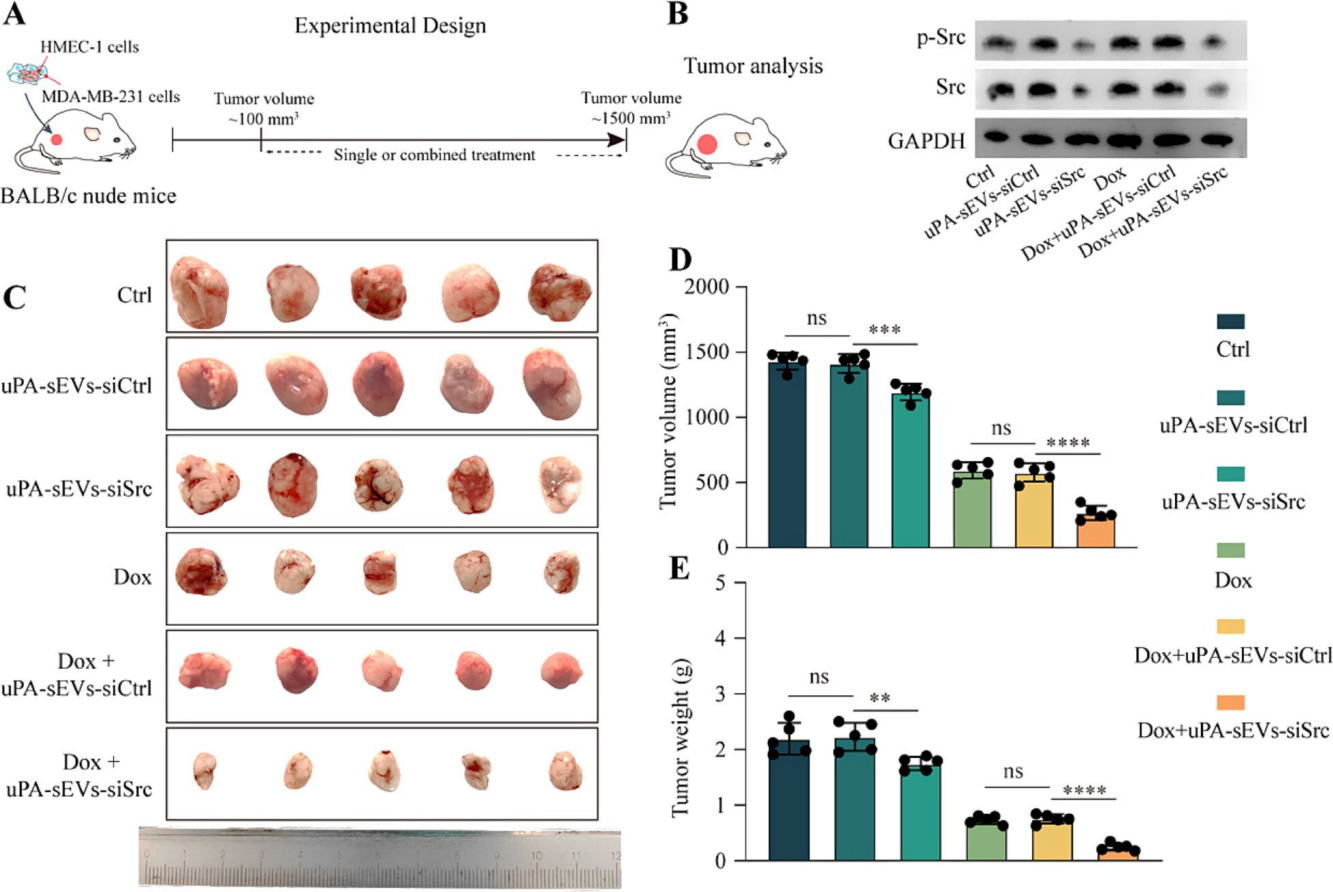
In vivo anti-tumor activity of uPA-sEVs-siSrc. (**A**) Schematic illustration describing the experimental design. (**B**) p-Src and Src protein expression in tumors with different treatment. (**C**) Representative images of tumor tissues with different treatment. (**D**) Volume of tumor tissues with different treatment. (**E**) Weight of tumor tissues with different treatment. ns, no significance; ^**^p<0.01; ^***^p<0.001; ^****^p<0.0001

To evaluate the anti-tumor activity of uPA-sEVs-siSrc in vivo, we generate tissue recombinants of tumor cells (MDA-MB-231) with stomal cells (HMEC-1) in a pre-optimized ratio (4:1) as previously described [47]. The cells were then subcutaneously implanted into the hind flanks of BALB/c nude mice to establish a tumor xenograft model of human breast cancer. When tumor volumes reached 50–100 mm^3^, the mice were randomly divided into six groups for the following treatments: (1) Control, (2) uPA-sEVs-siCtrl, (3) uPA-sEVs-siSrc,4) DOX, 5) DOX + uPA-sEVs-siCtrl, 6) DOX + uPA-sEVs-siSrc. The tumor tissues were analyzed when maximum tumor volume reached 1500 mm^3^ (Fig. 7A). As shown in Fig. 7B, uPA-sEVs-siSrc alone or combined with DOX treatment can significantly reduce the p-Src and total Src expression in the tumor tissues, compared to control and uPA-sEVs-siCtrl groups (Fig. 7B). We next examined the effects of uPA-sEVs-siSrc alone or in combination with DOX treatment on the tumor growth. uPA-sEVs-siSrc alone resulted in tumour shrinkage compared to the control and uPA-sEVs-siCtrl groups. DOX administration significantly delayed tumor growth, confirming the efficacy of DOX as a chemotherapeutic agent. Notably, treatment with DOX followed by uPA-sEVs-siSrc significantly enhanced tumor regression compared to DOX alone or DOX combined with uPA-sEVs-siCtrl (Fig. 7C-E).

**Fig. 8:**
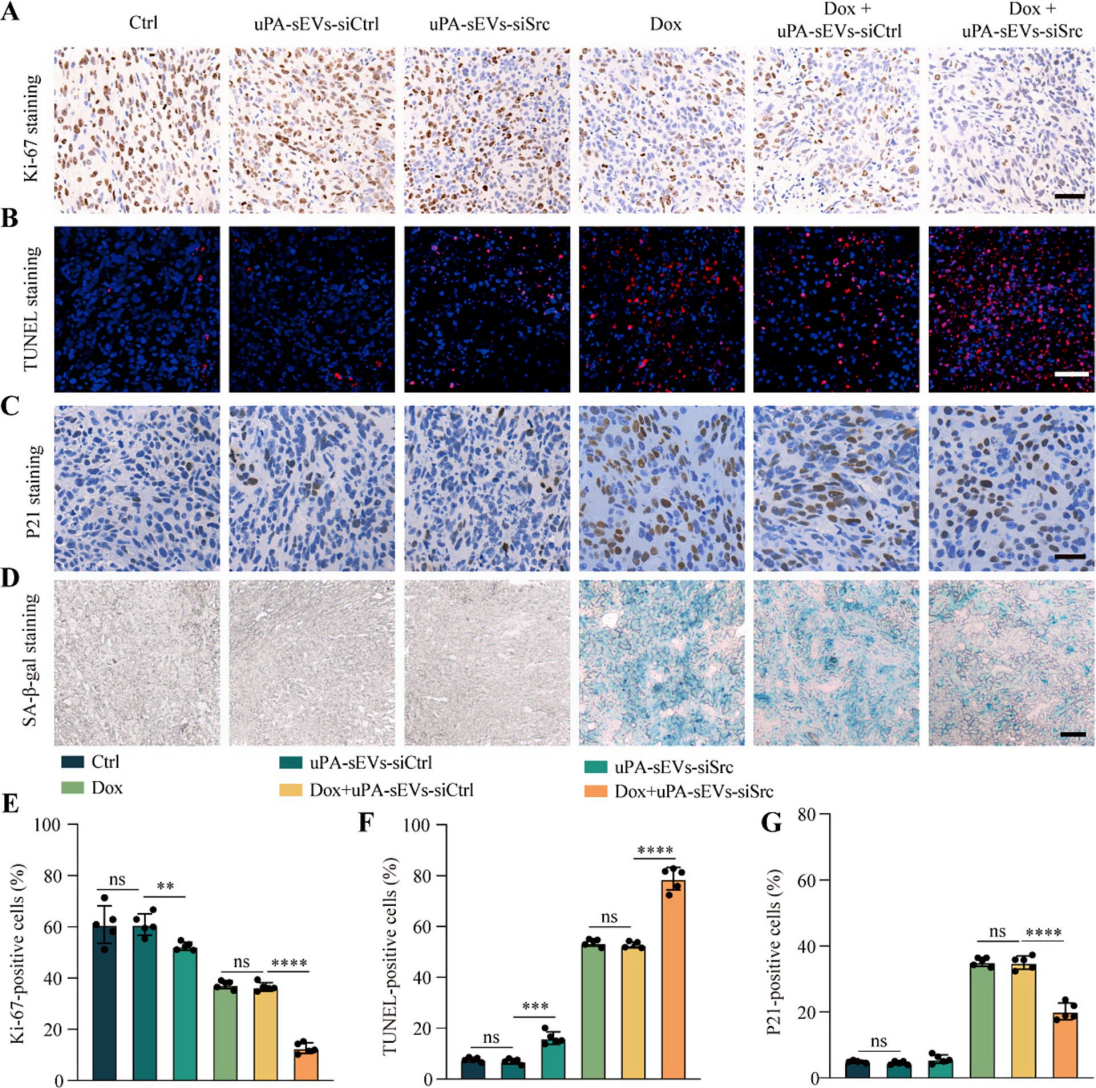
Histological analysis of tumor tissues. (**A**) Representative Ki-67 staining images of tumors collected from mice with different treatment. Scale bar = 100 μm. (**B**) Representative TUNEL staining images of tumors collected from mice with different treatment. Scale bar = 100 μm. (**C**) Representative P21 staining images of tumors collected from mice with different treatment. Scale bar = 100 μm. (**D**) Representative SA-β-gal staining images of tumors collected from mice with different treatment. Scale bar = 90 μm. (**E**) Quantification of the percentage of Ki-67 positive cells. (**F**) Quantification of the percentage of TUNEL positive cells. (**G**) Quantification of the percentage of P21 positive cells. ns, no significance; ^**^p<0.01; ^***^p<0.001; ^****^p<0.0001

Consistently, Ki-67 staining of tumor tissues showed that uPA-sEVs-siSrc alone treatment reduced the percentage of Ki-67-positive cells in tumor tissues compared to control and uPA-sEVs-siCtrl group. DOX administration significantly decreased Ki-67-positive cells, and treatment with DOX followed by uPA-sEVs-siSrc resulted in a much lower proportion of Ki-67-positive cells in the tumor tissues (Fig. 8A, E). Similarly, uPA-sEVs-siSrc treatment increased the TUNEL-positive cells in the tumor tissues compared to control and uPA-sEVs-siCtrl groups. Moreover, TUNEL-positive cells were more abundant in mice receiving DOX than in the control or uPA-sEVs-siSrc groups, but treatment with DOX followed by uPA-sEVs-siSrc resulted in an even higher proportion of TUNEL-positive cells within the tumor tissues (Fig. 8B, F). These results suggest that uPA-sEVs-siSrc treatment can promote tumor regression and enhance chemotherapy efficacy in vivo. The sections were also stained with markers of senescent cells. As shown in Fig. 8C, G, treatment with uPA-sEVs-siSrc alone did not increase the number of P21-positive cells in the tumor tissue compared to the control group. Meanwhile, DOX administration significantly increased the percentage of P21-positive cells, but treatment with DOX followed by uPA-sEVs-siSrc significantly decreased the percentage of P21-positive cells in tumor tissues. Consistently, SA-β-gal staining of tumor tissues showed that uPA-sEVs-siSrc treatment alone did not increase SA-β-gal-positive cells in tumor tissues compared to the control group. Treatment with DOX followed by uPA-sEVs-siSrc can significantly decrease the percentage of SA-β-gal-positive cells in tumor tissues compared to the DOX-treated group (Fig. 8D). These results indicate that uPA-sEVs-siSrc treatment can significantly reduce DOX-induced senescent cells in vivo.

With uPA-sEVs-siSrc displaying satisfying therapeutic strength, its toxicity was evaluated as safety is an essential concern for clinical use. From the H&E-stained sections, no histological changes are identifiable between control group and uPA-sEVs-siSrc group in heart, liver, spleen, lung, and kidney (Figure S8A). In addition, the levels of AST and ALT in the uPA-sEVs-siSrc group were similar to those in the control group (Figure S8B). These results suggest that uPA-sEVs-siSrc has low systemic toxicity in vivo.

## Conclusion

In summary, we constructed a versatile engineered sEVs platform named uPA-sEVs-siSrc. uPA-sEVs-siSrc can not only target and effectively induce apoptosis of tumor cells, but also simultaneously target and eliminate senescent stromal cells. uPA-sEVs-siSrc alone significantly inhibited tumor growth. Furthermore, uPA-sEVs-siSrc in combination with chemotherapeutic agents can significantly reduce DOX-induced senescence and enhance the inhibition of tumor growth by chemotherapeutic agents. To our knowledge, this study provides the first evidence that uPA-sEVs-siSrc can simultaneously target and eliminate senescent stromal cells and tumor cells. This engineered sEVs platform may serve as a promising therapy to kill two birds with one stone, promoting tumor regression and enhancing the efficacy of chemotherapeutic agents in tumor regression.

## Materials and methods

### Cell culture and characterization

The generation of mesenchymal stem cells from human induced pluripotent stem cells (human iPSC-MSCs, iMSC) has been described previously [34]. iMSCs were cultured under serum-free conditions using ncMission hMSC medium (Nuwacell Biotechnologies Co., Ltd, China, RP02010) and continuously passaged after reaching 90% confluence. Cells from 5 to 10 passages were used for subsequent experiments. The surface antigens of the iMSC were analyzed by flow cytometry as previously described [34]. Briefly, the iMSCs were stained with the following monoclonal antibodies (BD Bioscience): CD45, CD133, HLA-DR, CD44, CD29, CD146 and CD105, and analyzed using a CytoFLEX flow cytometer (Beckman Coulter Life Science, USA). Human lung cancer A549 cells and breast cancer MDA-MB-231 cells were cultured in Dulbecco’s modified Eagle’s medium (DMEM; Gibco) supplemented with 10% fetal bovine serum (FBS). Human foreskin fibroblasts-1 (HFF-1) were cultured in DMEM supplemented with 10% FBS, sodium pyruvate and non-essential amino acids. Human microvascular endothelial cell line-1 (HMEC-1) was cultured in MCDB131 medium supplemented with 10% FBS, epidermal growth factor (EGF) and L-glutamine (L-Glu). These cells were cultured at 37℃ with 5% CO_2_.

### Isolation and characterization of iMSC-sEVs

iMSC-sEVs were isolated by serial centrifugation with ultracentrifugation as previously described [48]. Briefly, conditioned medium (CM) was collected during the subculture process. All centrifugation steps were performed at 4℃. First, 500 mL of CM was collected and centrifuged at 300 × g for 5 min to pellet and remove dead cells. The supernatant was then spun at 2,000 × g for 20 min to remove debris and apoptotic bodies. The supernatant was then centrifuged at 10,000 × g for 30 min to pellet large EVs (IEVs). Afterwards, the media supernatant was passed through a 0.22 μm pore PES filter (Millipore, SCGPU05RE) to further remove any remaining IEVs. The supernatant was then subjected to ultracentrifugation at 100,000 × g for 70 min in an SW 32 Ti Rotor Swinging Bucket rotor (*k* factor of 256.8, 28,536 rpm, Beckman Coulter, Fullerton, CA) to pellet the iMSC-sEVs. The iMSC-sEVs pellet was resuspended in a large volume of PBS followed by ultracentrifugation at 100,000 × g for 70 min in the SW 32 Ti Rotor Swinging Bucket rotor to wash the sample. After PBS washing, the pellet of sEVs enriched fraction was resuspended in 200 µL PBS and stored at -80° C. Size distribution and particle concentration were measured by nanoscale flow cytometry (nanoFCM). Protein concentration was quantified using the Pierce BCA Protein Assay Kit according to the product manual. The morphology of sEVs was observed by transmission electron microscopy (TEM, TF20, FEI, USA).

### Preparation of uPA peptide modified and sisrc-loaded iMSC-sEVs (uPA-sEVs-siSrc)

DSPE-PEG-uPA (VSNKYFSNIHWGC) were synthesized at QYAOBIO (Shanghai, China), 10 µL iMSC-sEVs and 90 µL of the DSPE-PEG-uPA (10 µM) were added to 100 µL PBS, and the mixture was incubated overnight at 4℃. To remove unbound DSPE-PEG-uPA, the mixture was washed with PBS by ultracentrifugation and resuspended in sterile PBS. siRNA was loaded into iMSC-sEVs by electroporation using a Gene Pulser Xcell (BIO-RAD, USA). Briefly, uPA-sEVs and siRNA were gently mixed at a 1:1 (v/v) ratio in the prepared electroporation solution and then rapidly electroporated in a 2 mm cuvette at 400 mV and 125 µF capacity to obtain siRNA-loaded sEVs. To remove free siRNA, the mixture was washed with cold PBS by ultracentrifugation and resuspended in sterile PBS.

### Characterization of uPA-sEVs and uPA-sEVs-siSrc

The size distribution of uPA-sEVs and uPA-sEVs-siSrc was measured by using nanoFCM. The morphology was observed under TEM (TF20, FEI, USA). To investigate the storage stability of sEVs, the uPA-sEVs-siSrc was kept in PBS containing 10% FBS at 4℃ and measured by nanoFCM over consecutive 7 days. To determine the protection of sEVs on siRNA, the serum stability of the siRNA loaded in sEVs was evaluated by gel electrophoresis. Free siRNA, sEVs-siSrc or uPA-sEVs-siSrc were incubated with 10% FBS. Equal amounts of samples for each group of different incubation times were electrophoresed on agarose gels and then visualized with a UV illuminator. The unencapsulated siSrc in the supernatant was detected at 260 nm using a microplate reader, to calculate the siRNA loading of sEVs through the ratio of encapsulated siSrc to siSrc-loaded sEVs. DSPE-PEG-uPA conjugated with fluorescein FITC was synthesized. FITC-labelled uPA-sEVs and uPA-sEVs-siSrc were prepared and analyzed using nanoFCM. The zeta potential was analyzed by a Delsamax Pro nanoparticle analyzer (Beckman Coulter, USA). The antibodies against the following proteins were used for western blot analysis: CD9 (1:1000; ab92726, Abcam), CD63 (1: 1000; ab134045, Abcam), TSG101 (1: 1000; sc-7964, Santa cruz), Calnexin (1: 1000; 2679T, CST). Anti- rabbit IgG or anti-mouse IgG, HRP-linked antibody (1: 2,000; Cell Signaling Technology) was used as secondary antibody.

### Cellular uptake and lysosome escape of uPA-sEVs-siSrc *in vitro*

Senescent HFF-1 and HMEC-1 cells were induced by DOX treatment. In brief, HFF-1 and HMEC-1 cells were grown to 60~80% confluence and treated with DOX (100 nM, Selleck) for 72 h. After treatment, cells were rinsed twice with PBS and maintained in medium for 4–5 days. To evaluate the cellular uptake of uPA-sEVs-siSrc, stromal cells (HFF-1 and HMEC-1) and tumor cells (A549 and MDA-MB-231) were seeded in 96-well plates and incubated with DiI-labelled sEVs or uPA-sEVs-siSrc (1 × 10^9^ particles) for 4 h. The cells were then fixed in 4% paraformaldehyde and stained with DAPI, followed by imaging by using fluorescence microscope (DMi8, Leica). For flow cytometry analysis, the cells were washed with PBS twice and suspended with PBS before they were detected by flow cytometry (Cytoflex, USA). Stromal cells and tumor cells were treated with DiI-labeled uPA-sEVs-siSrc for different time (2 h, 8 and 16 h) to explore the endosome escape ability. The cells were rinsed and stained with lysotracker-green (Beyotime), which were observed under fluorescence microscope.

### Transfection efficiency and cytotoxicity of uPA-sEVs-siSrc

Proliferative or senescent stromal cells (HFF-1 and HMEC-1) and tumor cells (A549 and MDA-MB-231) were seeded in 12-well plates. Free scrambled siRNA, free siSrc, uPA-sEVs-siCtrl and uPA-sEVs-siSrc with a siRNA concentration of 100 nM and uPA-sEVs were added to each well. After incubation for 48 h, total RNA was extracted from cell pellets using TRIzol regent (Invitrogen, USA) according to the manufacturer’s instructions. Reverse transcriptase was performed using the RevertAid First Strand cDNA Synthesis Kit (Thermo Scientific, CA). PCR reactions were performed on the ABI Prism 7900HT Real Time System (Applied Biosystems, Carlsbad, CA) with SYBR green (Roche Applied Science). The primers of Src gene were listed as follows: Forward: GAGCGGCTCCAGATTGTCAA; Reverse: CTGGGGATGTAGCCTGTCTGT. Protein expression of p-Src (CST;6943T) and Src (CST; 2109T) was measured by western blot analysis.

Proliferative or senescent stromal cells (HFF-1 and HMEC-1) and tumor cells (A549 and MDA-MB-231) were seeded into wells of a 96-well plate. Cells were treated with scrambled siRNA, free siSrc, uPA-sEVs, uPA-sEVs-siCtrl and uPA-sEVs-siSrc for 48 h. Then the CCK-8 assay was then performed to assess cell viability according to the manufacturer’s instructions. Caspase-3 activity was measured using Caspase-3 Activity Assay kit (Beyotime Biotechnology, China) according to the manufacturer’s protocol. Apoptosis was also assessed using an Annexin V-FITC cell apoptosis assay kit (Beyotime Biotechnology, China). Briefly, cells were trypsinized, harvested, and incubated with Annexin V-FITC and PI before sorting using a Beckman flow cytometer (Beckman).

### Senescence-associated β-galactosidase (SA-β-gal) assay and immunofluorescence (IF) staining

SA-β-gal staining of stromal cells was performed using an SA-β-gal staining kit (Beyotime Biotechnology, China) according to the manufacturer’s protocol. In brief, cells were fixed in 4% paraformaldehyde for 5 min. After washing with PBS three times, samples were incubated in SA-β-gal solution at 37 °C overnight. Ice-cold PBS was then used to stop the enzymatic reaction. In blinded analyses, for each sample, 10 images were taken from random fields using microscope. The ratio of positive cells was determined by counting the blue cells and dividing by the total number of observed cells.

For IF staining, stromal cells were fixed with 4% paraformaldehyde, permeabilized in PBS containing 0.5% Triton X-100 for 15 min at room temperature and pre-incubated with 5% BSA for 1 h at room temperature to block non-specific staining. Fixed cells were then incubated over-night at 4 °C with primary antibodies against P21 (1:500; ab188224, Abcam), γ-H2AX (1:400; 9718s, Cell Signaling Technology), followed by secondary antibody conjugated to Alexa Fluor 594 (1:1000; Thermo Scientific). Nuclei were labelled with DAPI (1:1000; D9542, Sigma) at room temperature for 5 min. Fluorescence images were captured under fluorescence microscope (Leica Microsystems). Quantification of the number of positively stained cells was performed by using the ImageJ software.

### Biodistribution and anti-tumor efficacy study of uPA-sEVs-siSrc **in vivo**

Animal care and experimental procedures were approved by the Animal Research Committee of the Shanghai Jiao Tong University Affiliated Sixth People’s Hospital (DWSY2021-0155) and were in accordance with the Guide for the Care and Use of Laboratory Animals published by the US National Institutes of Health (NIH publication, 8th edition, 2011). BALB/c nude mice aged 6–8 weeks were housed under pathogen-free conditions and provided with standard diet and water. At the end of the experiment, the mice were euthanized with an overdose of pentobarbital sodium.

MDA-MB-231 and HMEC-1 cells were mixed at a ratio of 1:4, with each in vitro recombinant containing 1 × 10^7^ total cells prior to subcutaneous implantation. When tumor volumes reached approximately 100 mm^3^, PKH26-labelled sEVs and uPA-sEVs-siSrc were injected intravenously into tumor-bearing mice. Mice were imaged using the IVIS Spectrum system 8 h after injection, and major organs (heart, liver, spleen, lung, kidney) and tumors were harvested for ex vivo imaging. To investigate the distribution of uPA-sEVs-siSrc in tumor tissues, the tissue samples were sectioned into 10 μm thick slices, followed by fixation and DAPI staining. Fluorescence signals were observed under a fluorescence microscope (DMi6, Leica).

Subcutaneous xenografts were established by implanting MDA-MB-231 and HMEC-1 recombinant cell suspension (1 × 10^7^ cells) into the BALB/c nude mice at 6–8 weeks of age. When tumor volumes reached 50–100 mm^3^, the mice were randomly divided into six groups for the following treatments: (1) Control, (2) uPA-sEVs-siCtrl (1 × 10^10^ particles in 100 µL PBS, tail vein injection, twice weekly), (3) uPA-sEVs-siSrc (1 × 10^10^ particles in 100 µL PBS, tail vein injection, twice weekly), (4) DOX (4 mg/kg body weight, i.p., twice weekly), (5) DOX + uPA-sEVs-siCtrl, (6) DOX + uPA-sEVs-siSrc. The tumor volume and body weight of the mice were monitored over time. The tumor volume was calculated as follows: volume=(length × width^2^)/2. Excised tumors were either freshly snap-frozen or fixed in 4% PFA and then processed for immunofluorescence staining.

### Histological evaluation and toxicity analysis

For immunohistochemical staining, the formalin-fixed tumor tissues were cut into Sect. (5 μm thickness) and performed with Ki67 and P21 staining. TUNEL assay was performed following the manufacturer’s instructions. For SA-β-gal staining of tumor tissues, frozen sections were dried at 37℃ for 20–30 min before being fixed for 15 min at room temperature. Frozen sections were washed three times with PBS and incubated with SA-β-gal staining solution (Beyotime Biotechnology, China) overnight at 37℃. After completion of SA-β-gal staining, sections were rinsed under running water for 1 min. After drying, samples were examined under a bright-field microscope. The hematoxylin-eosin (H&E) staining of heart, liver, spleen, lung, and kidney was performed to evaluate the toxicity of uPA-sEVs-siSrc in vivo. Whole blood (*n* = 5 for each group) was collected and plasma was isolated to measure representative blood parameters including alaninetransaminase (AST) and alanine aminotransferase (ALT) by AST and ALT activity kits.

### Statistical analysis

Numerical data are presented as the mean ± SD. One-way ANOVA for three or more groups and Student’s t-test for two groups were performed for statistical analysis using GraphPad Prism 7.0.

### Electronic supplementary material

Below is the link to the electronic supplementary material.

Supplementary Material 1

## Data Availability

All data are available in the main text and are available from the corresponding authors upon reasonable request.
