# Supplementary material for: A versatile engineered extracellular vesicle platform simultaneously targeting and eliminating senescent stromal cells and tumor cells to promote tumor regression
Source: J Nanobiotechnology. 2024 Mar 11;22:105. doi: 10.1186/s12951-024-02361-3 (PMC10926582; doi:10.1186/s12951-024-02361-3)
Supplement: Supplementary file 1 — Supplementary Material 1 [file 12951_2024_2361_MOESM1_ESM.docx]

**A versatile engineered extracellular vesicle platform simultaneously targeting and eliminating senescent stromal cells and tumor cells to promote tumor regression**

Liangzhi Gong^1#^, Zhengsheng Chen^1#^, Kai Feng^1#^, Lei Luo^1^, Juntao Zhang^1^, Ji Yuan^1^, Yajing Ren^1^, Yang Wang^1*^, Xianyou Zheng^1*^, Qing Li^1*^

^1^Institute of Microsurgery on Extremities, Department of Orthopedic Surgery, Shanghai Sixth People’s Hospital Affiliated to Shanghai Jiao Tong University School of Medicine

*Corresponding authors:

Qing Li, Institute of Microsurgery on Extremities, Department of Orthopedic Surgery, Shanghai Sixth People’s Hospital Affiliated to Shanghai Jiao Tong University School of Medicine, Shanghai 200233, China.

E-mail: [liqing_236@aliyun.com](mailto:liqing_236@aliyun.com)

Xianyou Zheng, Institute of Microsurgery on Extremities, Department of Orthopedic Surgery, Shanghai Sixth People’s Hospital Affiliated to Shanghai Jiao Tong University School of Medicine, Shanghai 200233, China.

E-mail: zhengxianyou@126.com

Yang Wang, Institute of Microsurgery on Extremities, Department of Orthopedic Surgery, Shanghai Sixth People’s Hospital Affiliated to Shanghai Jiao Tong University School of Medicine, Shanghai 200233, China.

E-mail: [wangyang63@sjtu.edu.cn](mailto:wangyang63@sjtu.edu.cn)

^#^Liangzhi Gong, Zhengsheng Chen and Kai Feng contributed equally to this work.


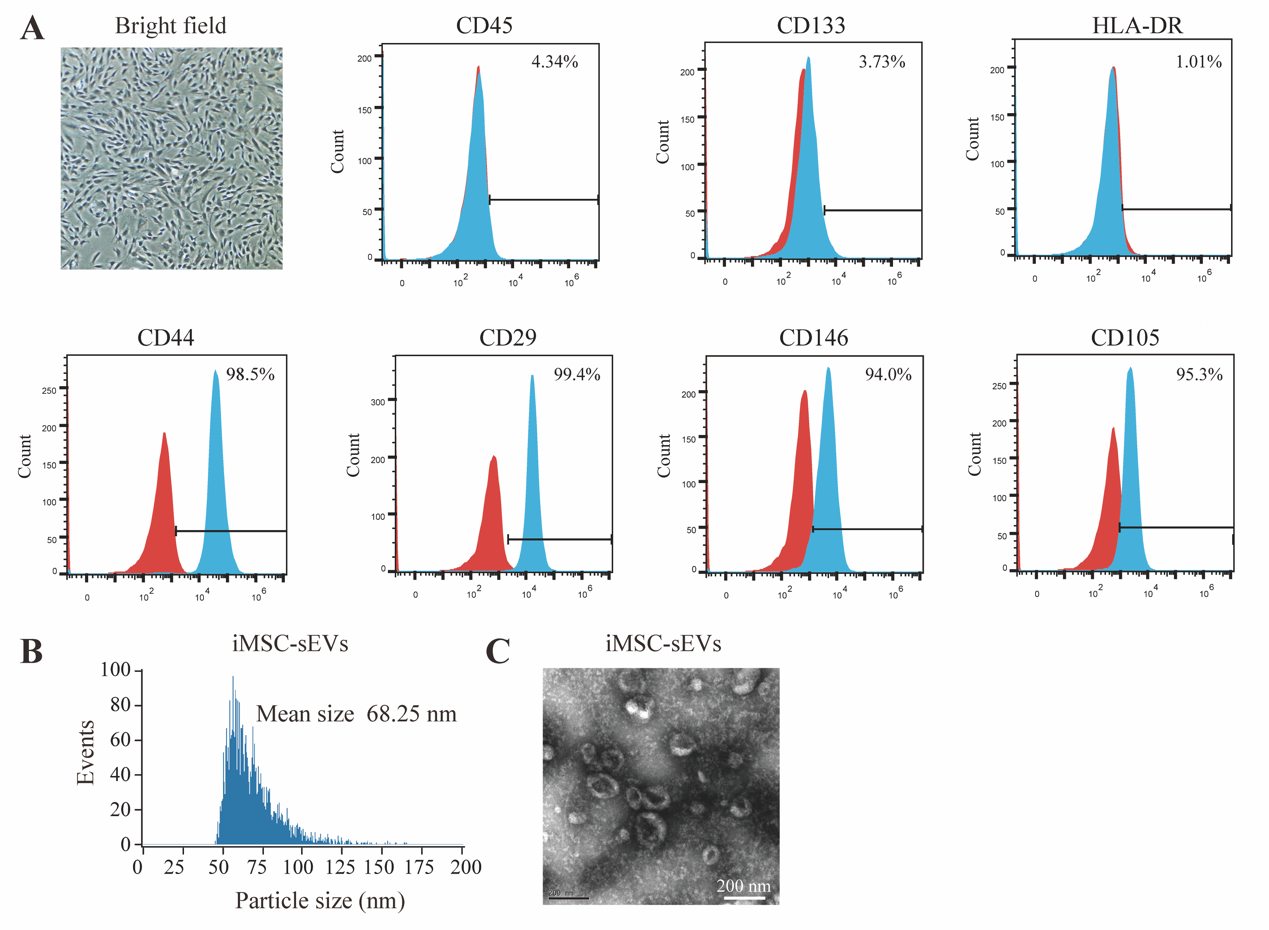


**Figure S1. Characterization of iMSC and iMSC-sEVs.** (A) The morphology of iMSC and the surface antigens of the iMSC analyzed by flow cytometry. (B) Size distribution of iMSC-sEVs measured by nanoFCM. (C) Representative TEM images of iMSC-sEVs. Scale bar = 200 nm.


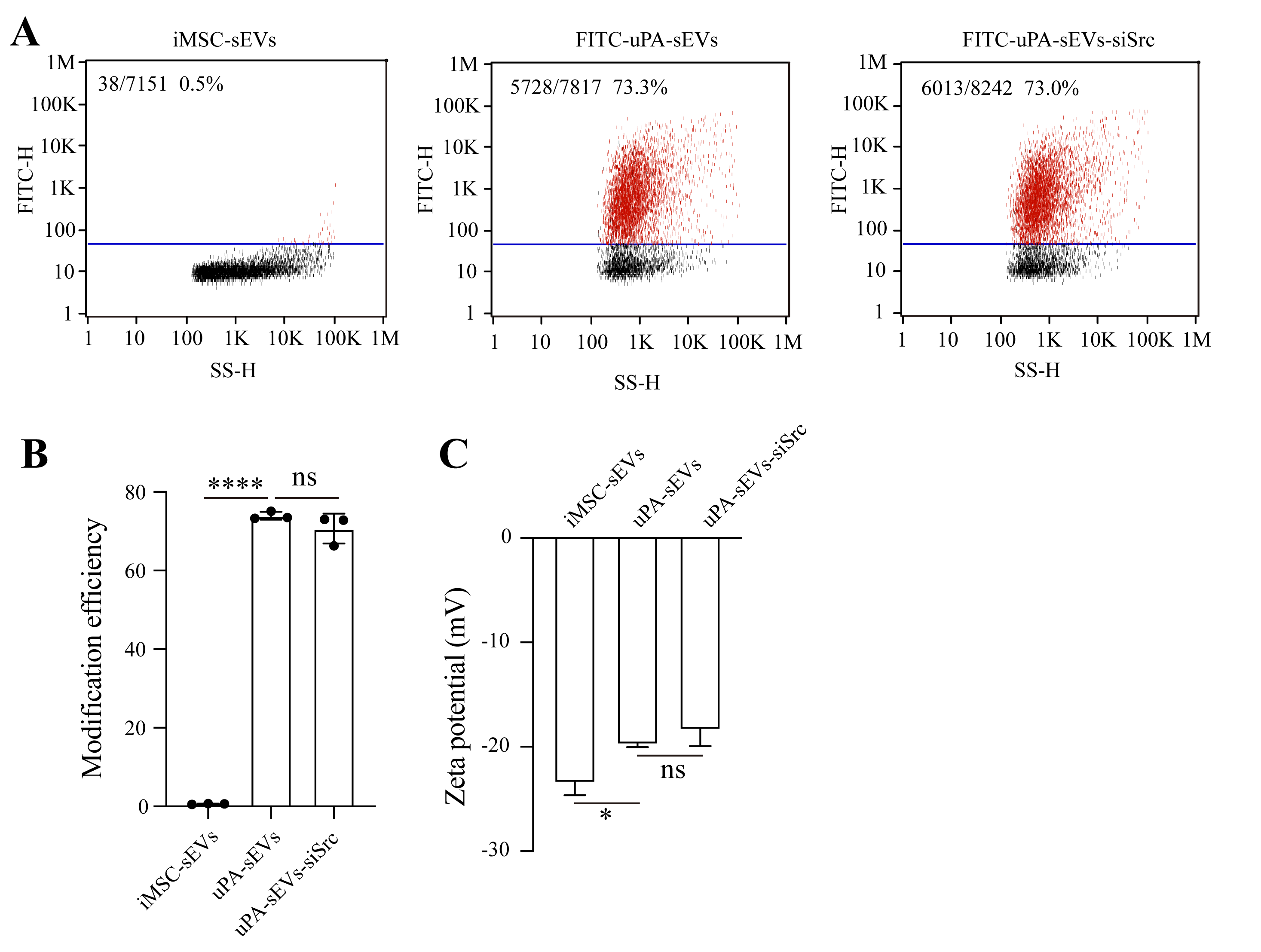


**Figure S2. Characterization of uPA-sEVs-siSrc.** (A) Modification of uPA peptide on iMSC-sEVs analyzed by nano-flow cytometry. (B) Quantitative analysis of modification efficiency (n=3). (C) Zeta potential of iMSC-sEVs, uPA-sEVs and uPA-sEVs-siSrc (n = 3). ^*^*p*＜0.05; ^****^ *p*＜0.0001.


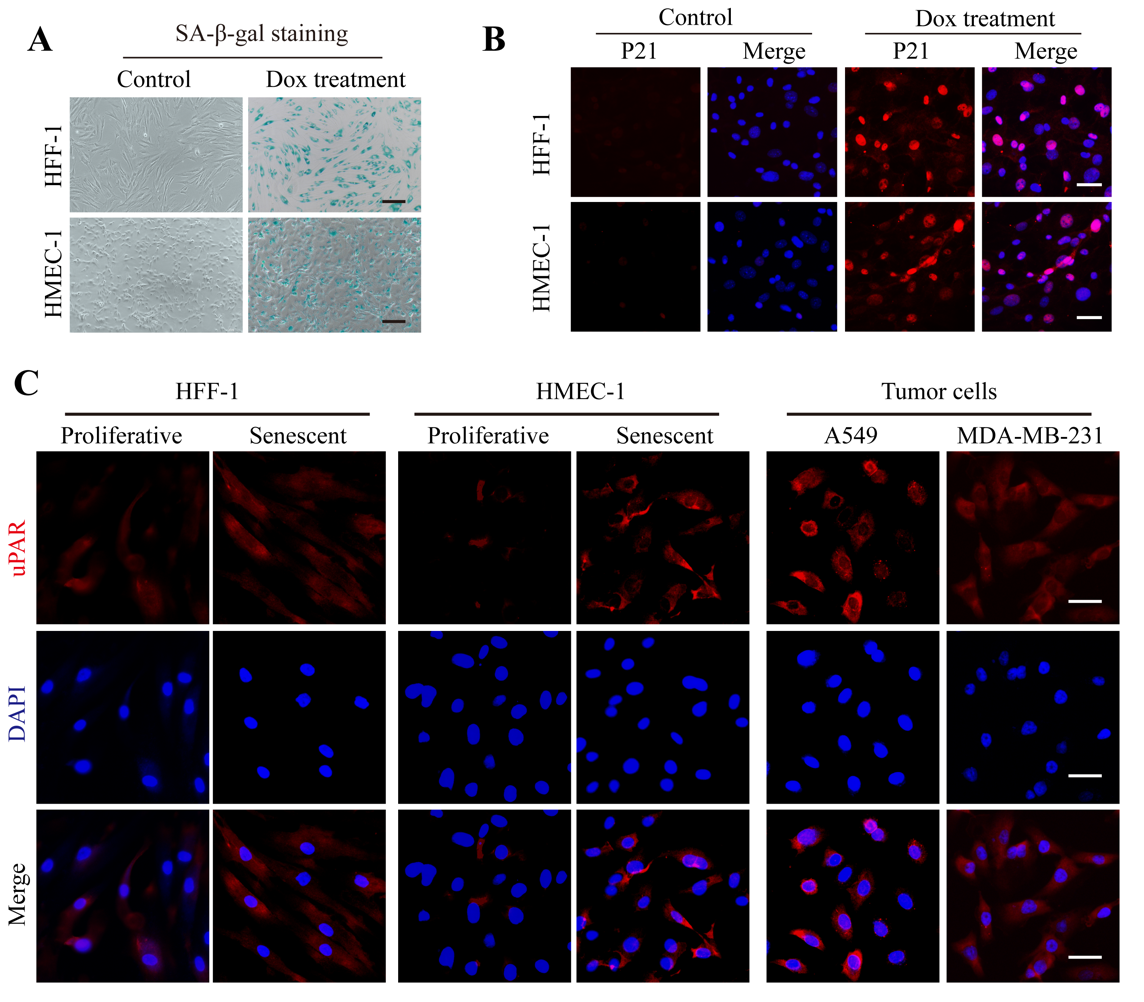


**Figure S3. Analysis of senescence phenotype and uPAR expression in doxorubicin-treated stromal cells.** (A) SA-β-gal staining of control and DOX-treated HFF-1 and HMEC-1 cells. Scale bar = 100 μm.(B) P21 staining of control and DOX-treated HFF-1 and HMEC-1 cells induced by doxorubicin. Scale bar = 50 μm. (C) Analysis of uPAR expression in proliferative and senescent stromal cells, and tumor cells. Scale bar = 50 μm.


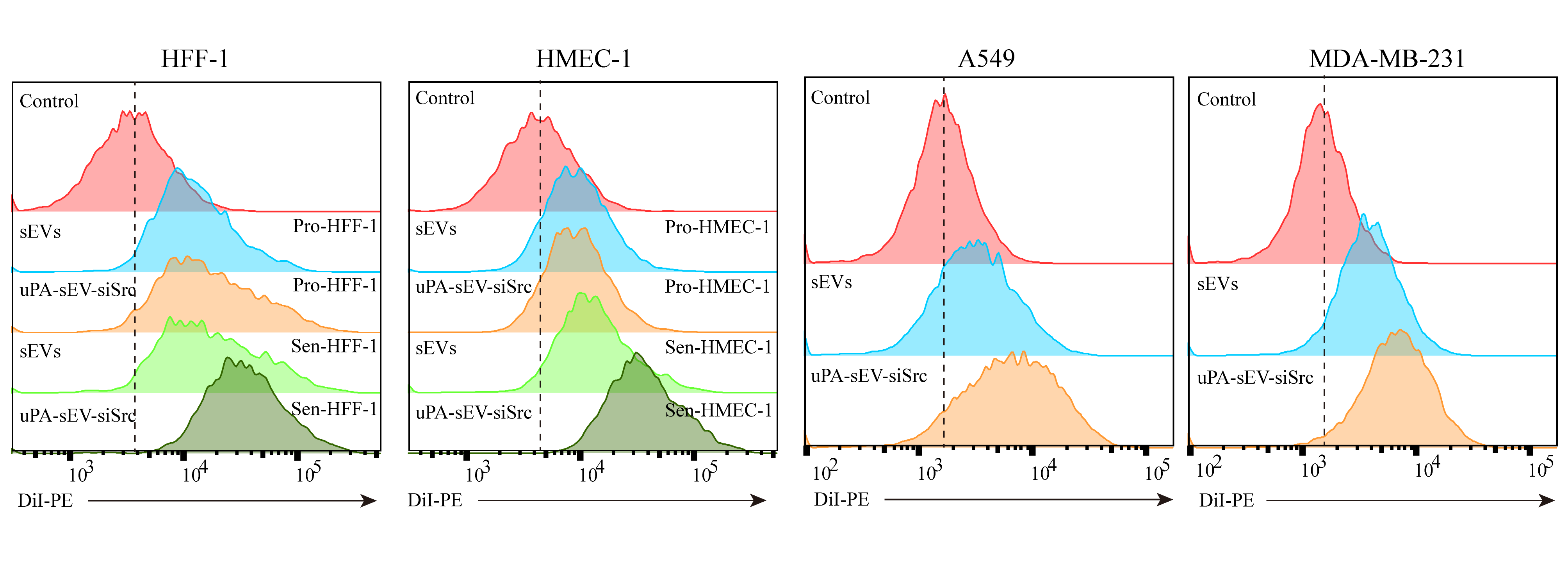


**Figure S4.** **Quantitative analysis of cellular uptake.** Uptake of DiI-labelled uPA-sEVs-siSrc or sEVs detected by flow cytometry in proliferative and senescent stromal cells and tumor cells.


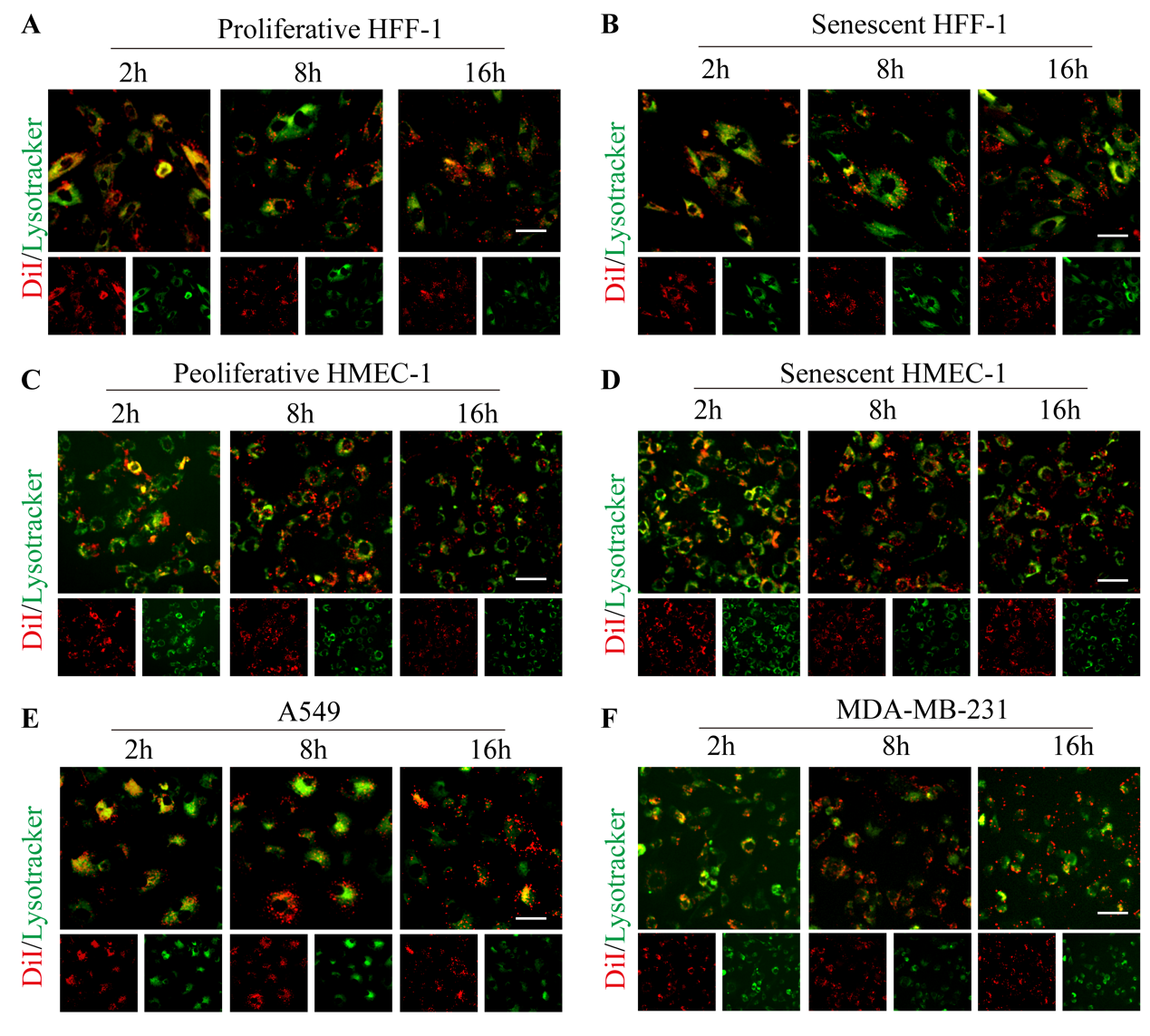


**Figure S5.** Representative images of cellular uptake of DiI-labeled uPA-sEVs-siSrc (Red) in proliferative and senescent HFF-1 cells (A, B), proliferative and senescent HMEC-1 cells (C, D), A549 cells (E) and MDA-MB-231 cells (F) stained with lysotracker (Green: lysosomes). Yellow color represented colocalization of fluorescent signals from sEVs and lysosomes. Scale bar = 50 μm.


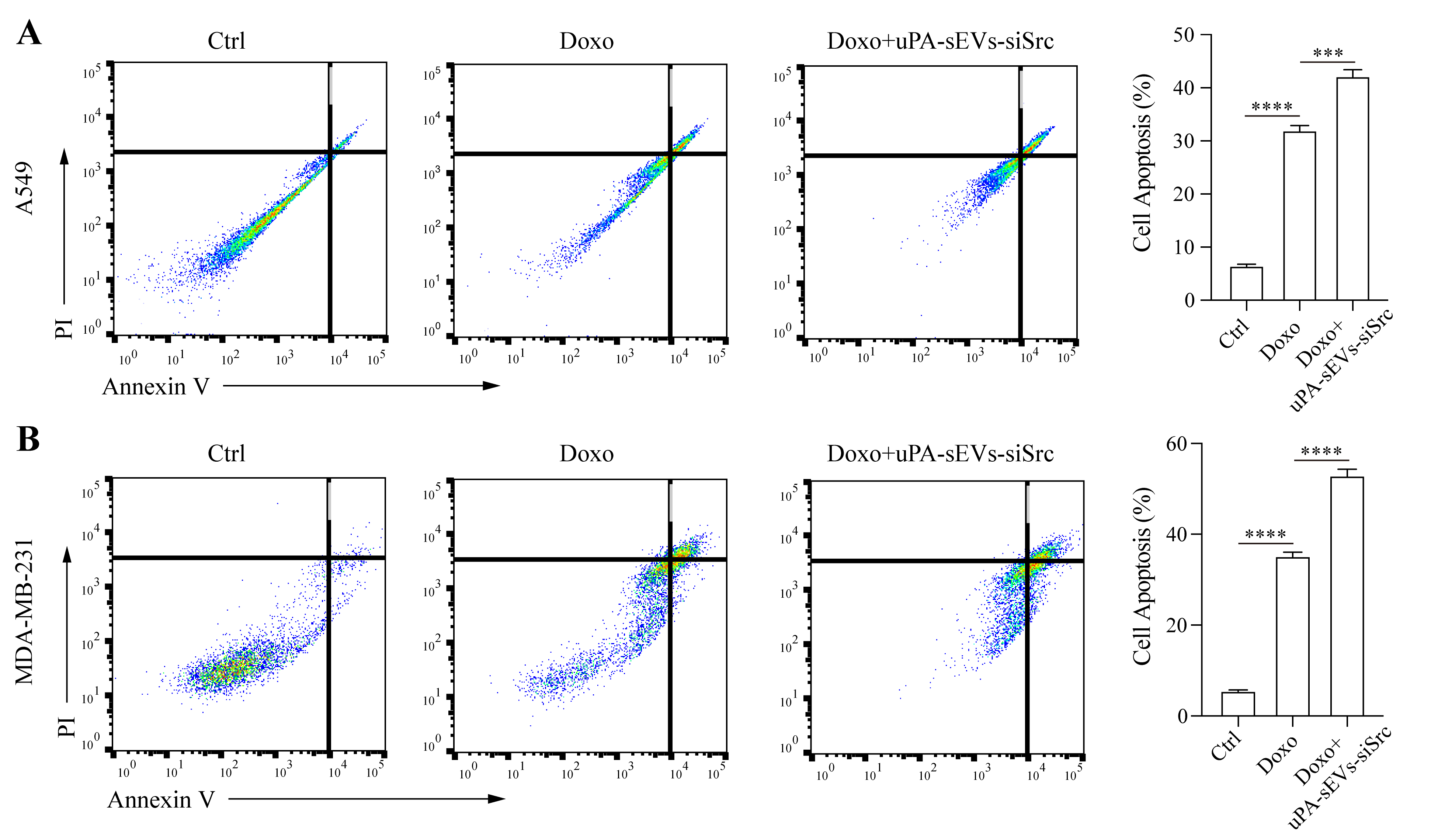


**Figure S6. uPA-sEVs-siSrc induce apoptosis of tumor cells *in vitro*.** Flow cytometry and quantitative analysis of apoptosis in A549 cells (A) and MDA-MB-231 cells (B) after treatment with DOX alone or in combination with uPA-sEVs-siSrc. Data are displayed as the mean ±SD (n=3). ^***^*p*＜0.001; ^****^ *p*＜0.0001.

**
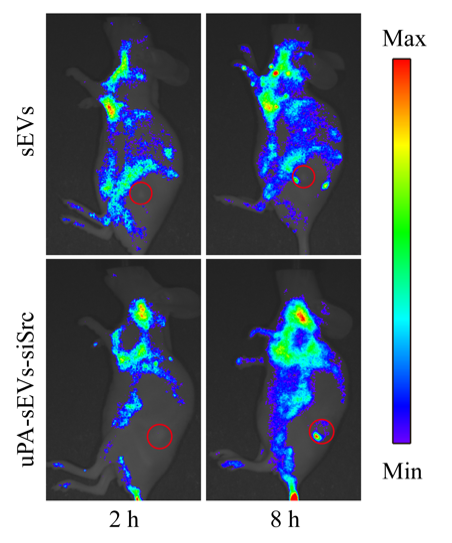
**

**Figure S7. Targeting ability of uPA-sEVs-siSrc *in vivo*.** *In vivo* fluorescent images of tumor-bearing mice after injection of PHK26-labelled sEVs and uPA-sEVs-siSrc. Tumor sites marked with red circles.


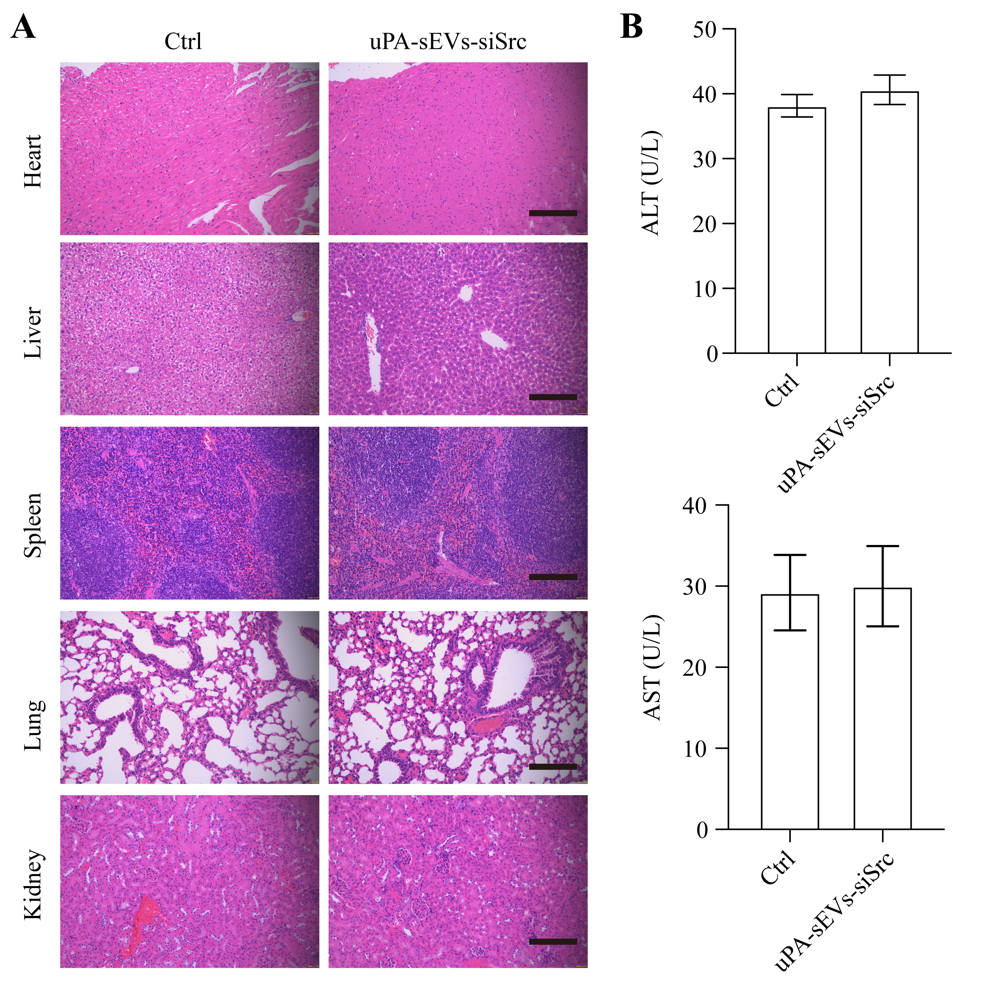


**Figure S8. Toxicity of uPA-sEVs-siSrc *in vivo*.** (A) H&E-stained sections of heart, liver, spleen, lung and kidney. Scale bar = 100 μm. (B) Serum measurement of ALT and AST at the end of therapeutic regimens.
